# Ethanol-triggered Lipophagy Requires SQSTM1 in AML12 Hepatic Cells

**DOI:** 10.1038/s41598-017-12485-2

**Published:** 2017-09-26

**Authors:** Lin Wang, Jun Zhou, Shengmin Yan, Guangsheng Lei, Chao-Hung Lee, Xiao-Ming Yin

**Affiliations:** 1 Department of Pathology and Laboratory Medicine, Indiana University School of Medicine, Indianapolis, IN 46202 USA; 2 College of Animal Science and Veterinary Medicine, Shandong Agricultural University, 61 Daizong Street, Tai’an City, Shandong Province 271018 China; 3 Shandong Provincial Key Laboratory of Animal Biotechnology and Disease Control and Prevention, Shandong Agricultural University, 61 Daizong Street, Tai’an City, Shandong Province 271018 China; 4 Shandong Provincial Engineering Technology Research Center of Animal Disease Control and Prevention, Shandong Agricultural University, 61 Daizong Street, Tai’an City, Shandong Province 271018 China; 5 Center of Minimally Invasive Surgery, Xiangya 2nd Hospital, Central South University, Changsha, Hunan 410011 China

## Abstract

Ethanol-induced hepatic lipophagy plays an important cytoprotective role against liver injury, but its mechanism is not fully determined. In the present study, ethanol-induced lipophagy was studied in an immortalized mouse hepatocyte line, AML12. We found that ethanol treatment elevated lipid content in these cells, which could be regulated by autophagy. To determine the potential mechanism, we investigated the role of a key adaptor molecule SQSTM1/p62. SQSTM1 can bind to LC3 on autophagosomes and ubiquitinated molecules on cargos, thus facilitating the autophagic engulfment of the cargo. We found that both LC3 and SQSTM1 could colocalize with lipid droplets (LDs) following ethanol treatment. Colocalization of LC3 with LDs was significantly inhibited by SQSTM1 knockdown, which also reduced ethanol-induced lipid elevation. In addition, increased ubiquitin signals were found to colocalize with SQSTM1 on LDs in response to ethanol. Moreover, the SQSTM1 signal was colocalized with that of perilipin1, a major protein on LDs. Finally, perilipin1 knockdown significantly altered ethanol-induced lipophagy. Taken together, these data support a model in which autophagosomes were directed to the LDs via SQSTM1, which bound to ubiquitinated proteins, possibly including perilipin 1, on LDs. This study provides a potential mechanistic explanation to how ethanol induces lipophagy in hepatocytes.

## Introduction

Alcoholic liver disease (ALD) is caused by chronic alcohol abuse and is a serious health concern worldwide. It is generally considered that the pathogenesis of ALD is intimately related to oxidative stress, derived from reactive intermediates including acetaldehyde, increased NADH/NAD^+^ ratio and reactive oxygen species (ROS) generation^1–4^. ALD is also characterized by an excessive accumulation of fatty acids. Free fatty acids can be detrimental to hepatocytes. An increased level of fatty acids and ROS can result in lipid peroxidation and increased production of inflammatory cytokines, contributing to liver injury and progression to fibrosis^5–10^.

In response to ethanol stimulation, cellular protection mechanisms including autophagy can be activated^5,6^. Autophagy is an evolutionarily conserved cellular degradation process with important pathophysiological significance^7^. Autophagy is particularly important for liver physiology and its disturbance has been well documented in liver diseases^8–10^. Deletion of key autophagy genes in the liver results in significant injury^11–14^. Autophagy can protect against liver injury by several mechanisms, among which is the ability of autophagy to eliminate intracellular lipids. Ethanol promotes lipid accumulation in lipid droplets (LDs). They may be considered less harmful than free fatty acids^15^. On one hand, de-esterification can occur, which would increase the cellular level of harmful free fatty acids. On the other hand, removal of lipid droplet may favor the equilibrium toward the esterification. Autophagy can reduce the level of LDs by lysosomal degradation. This process, known as lipophagy^16^, is still far from a complete understanding. Nevertheless, lipophagy occurs following ethanol stimulation. Colocalization of LC3 with lipid droplet can be demonstrated *in vivo* and *in vitro* ^5,6,17^. Hepatic triglycerides (TG) level in alcoholic fatty liver disease models was reduced by activating autophagy, and was elevated when autophagy was inhibited^5,6^.

However, the mechanism of lipophagy in ALD is not well understood regarding how it is initiated, and how it is regulated. A major hurdle in addressing these questions is the lack of proper cellular model to study how ethanol triggers autophagy stimulation in a controllable *in vitro* environment. This study intended to establish such a model system to study ethanol-induced lipophagy, which should provide a useful tool to study the initiation and regulation of ethanol-induced lipophagy. Using this model, we have also characterized the role of SQSTM1, a major autophagy adaptor, and perilipin 1, a major protein on lipid droplets, and found that both were important for ethanol-induced lipophagy.

## Results

### Ethanol-induced lipid accumulation in AML12 cells was regulated by autophagy

**Figure 1:**
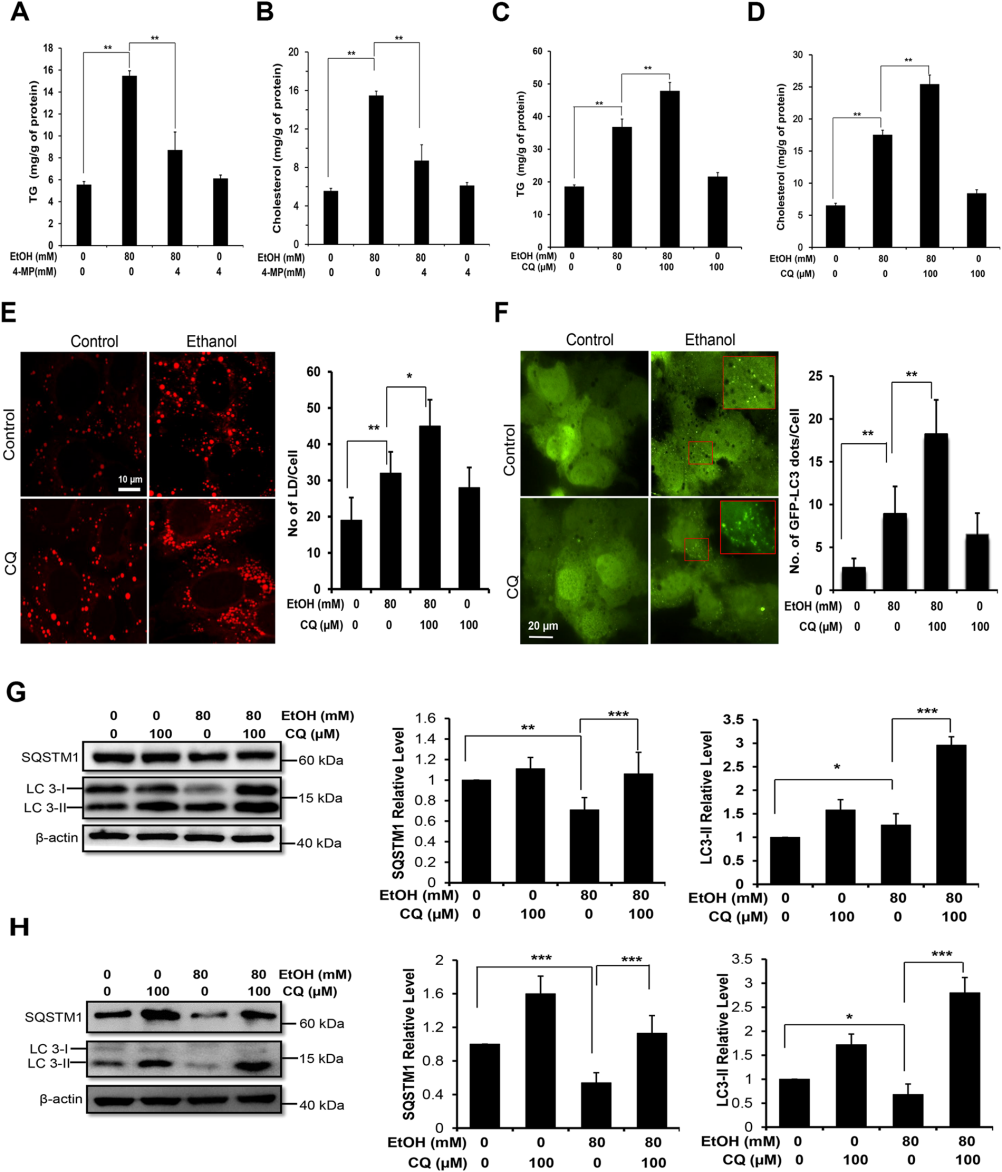
Ethanol-induced lipid accumulation is affected by ethanol metabolism and lysosome function. (**A**–**D**) AML12 cells were treated with ethanol for 24 hours in the presence or absence of 4-MP (**A**,**B**), or CQ (**C**,**D**). Intracellular levels of TG (**A**,**C**) and cholesterol (**B,D**) were then determined. (**E**) AML12 cells were incubated with ethanol (24 h) with or without CQ, and then stained with 1 µM Bodipy-581/591 for lipid droplets (LD), which were quantified. (**F**) AML12 cells were cultured on coverslips in 24-well plates, infected with adenoviral GFP-LC3 for 24 h, and then treated with ethanol for 24 hours with or without CQ. The number of GFP-LC3 puncta were quantified. Boxed areas are enlarged in the inserts, showing the GFP-LC3 puncta. (**G**,**H**) Cells were cultured with ethanol for 12 hours (**G**) or 24 hours (**H**) with or without CQ. Cell lysates were examined by immunoblotting for SQSTM1 and LC3. Densitometry was conducted, normalized to β-actin and expressed as fold of the control level. In all experiments, CQ treatment was only for the last three hours of the culture. Data represent mean ± SEM. ^*^ *P* < 0.05, ^**^ *P* < 0.01, ^***^ *P* < 0.001.

AML12 cells are responsive to ethanol because they retain the essential enzymes for ethanol metabolism^18^. The lipid content in AML12 cells as measured by the levels of TG and cholesterol was elevated in 24 hours after ethanol treatment (Fig. 1A and B). This elevation could be inhibited by 4-Methylpyrazole hydrochloride (4-MP), an inhibitor of alcohol dehydrogenase (Fig. 1A and B), indicating that the effect required the metabolism of ethanol^5,18,19^.

To determine whether ethanol-induced lipid accumulation in AML12 cells was affected by autophagy, we co-treated cells with chloroquine (CQ), which inhibits lysosome degradation. Indeed, CQ co-treatment further elevated the levels of TG and cholesterol stimulated by ethanol (Fig. 1C and D), suggesting that a part of lipids were normally removed from the cells through the lysosome-mediated degradation. We further confirmed the observation by examining the level of lipid droplets, which is the main form of the lipids in the ethanol-treated cells (Fig. 1E). To directly assess the activation of autophagy, we expressed in AML12 cells the autophagy marker LC3 conjugated to green fluorescence protein (GFP) and found that GFP-LC3 formed puncta upon ethanol treatment, indicating the formation of autophagosomes (Fig. 1F). Consistently, the autophagosome-bound form of LC3, known as LC3-II, was elevated, while the cytosol form of LC3, known as LC3-I, was reduced following ethanol treatment for 12 hours (Fig. 1G) or 24 hours (Fig. 1H). The level of SQSTM1/p62, an adaptor for autophagic targets, was reduced in ethanol-treated cells as the result of increased autophagic degradation (Fig. 1G and H). The enhanced autophagy flux was further confirmed by the co-treatment of CQ, which resulted in the highest elevation of GFP-LC3 puncta (Fig. 1F) or LC3-II (Fig. 1G and H), indicating the progression of autophagy through the lysosome-mediated degradation.

**Figure 2:**
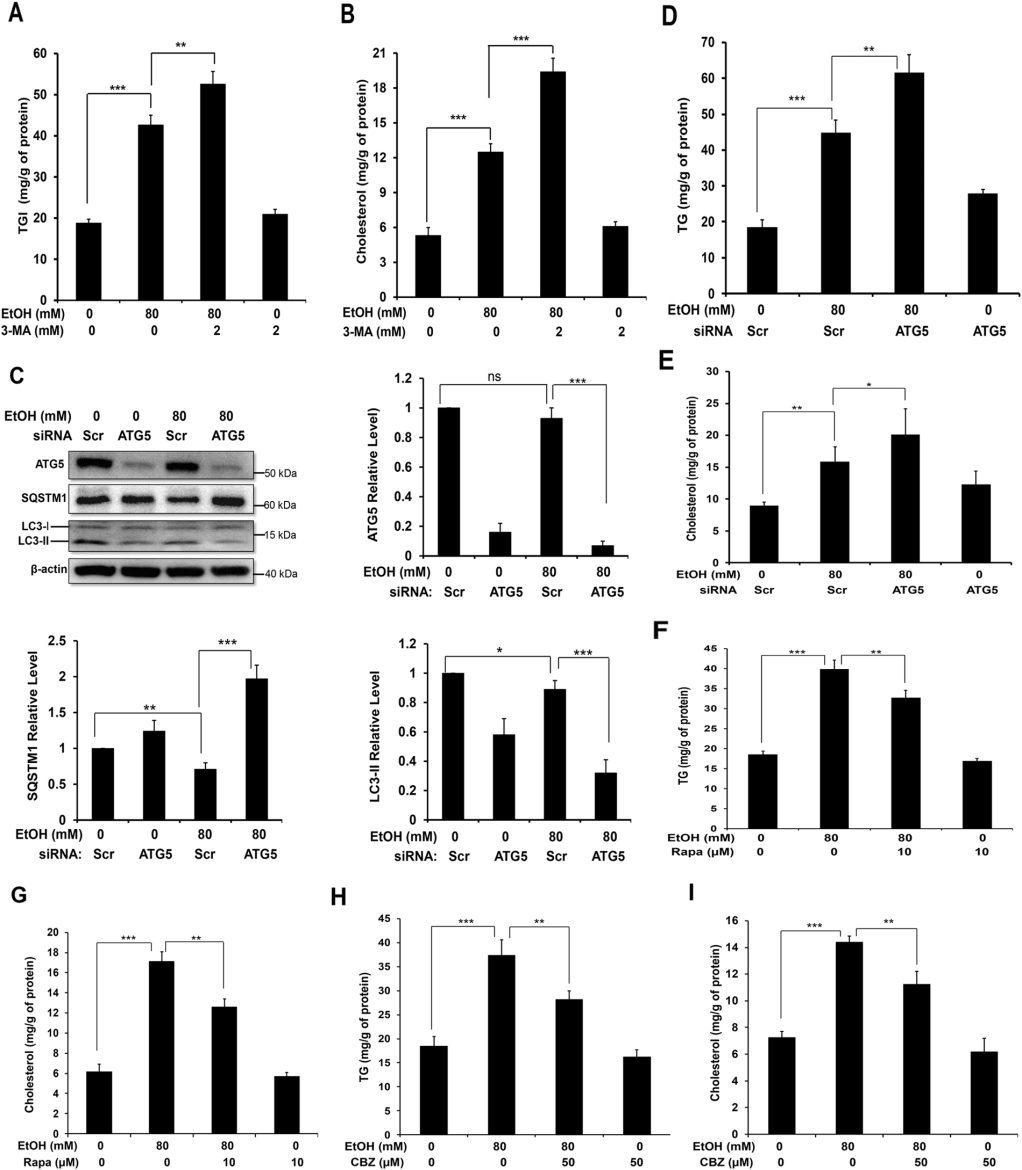
Modulation of autophagy affects the level of ethanol-induced lipids. (**A** and **B**) AML12 cells were simultaneously incubated with ethanol and 3-MA for 24 hours. Cells were then harvested and analyzed for intracellular levels of TG (**A**) and cholesterol (**B**). (**C**–**E**) AML12 cells were transfected with scramble (Scr) siRNA or ATG5-specific siRNA for 24 h, and then treated with ethanol for another 24 hours. Cells lysates were analyzed for levels of ATG5, SQSTM1, LC3, TG (**D**), and cholesterol (**E**). The protein levels were normalized to that of β-actin and expressed as fold of the control level. (**F**–**I**) Cells were incubated with ethanol in the presence or absence of rapamycin (**F** and **G**) or CBZ (**H**,**I**) for 24 hours. Intracellular levels of TG (**F,H**) and cholesterol (**G,I**) were determined. Data represent mean ± SEM. ^*^ *P* < 0.05, ^**^ *P* < 0.01, ^***^ *P* < 0.001.

We then determined whether directly altering the autophagy machinery could affect the level of lipids in ethanol-treated AML12 cells. The Beclin 1-Atg14 directed Class III PI-3 kinase complex plays a critical role in autophagy activation^20,21^. By inhibiting the PI-3 kinase with 3-methyladenine (3-MA) (Fig. 2A and B, Supplementary Fig. 1SA) or wortmannin (Supplementary Fig. 1SB–D), we found that ethanol-induced lipid accumulation was further elevated along with the suppression of LC3-II formation and SQSTM1 degradation. To more specifically assess the regulation of lipid content by autophagy under ethanol stimulation, we selectively knocked down a key autophagy gene, ATG5, in AML12 cells, which caused suppression of autophagy as indicated by the elevation of SQSTM1 and reduction of LC3-II (Fig. 2C). As in the case of co-treatment with CQ or PI-3 kinase inhibitors, knockdown of ATG5 led to a further accumulation of TG and cholesterol in ethanol-treated cells (Fig. 2D and E). Conversely, promotion of autophagy with known autophagy inducers, rapamycin (Supplementary Fig. 2SA) or carbamazepine (Supplementary Fig. 2SB), reduced the lipid content in ethanol-treated AML12 cells (Fig. 2F–I). Collectively, these data indicated that autophagy regulated the lipid content in AML12 cells following ethanol treatment.

### Autophagosomes and p62 were colocalized with lipid droplet, which is required for efficient lipophagy

**Figure 3:**
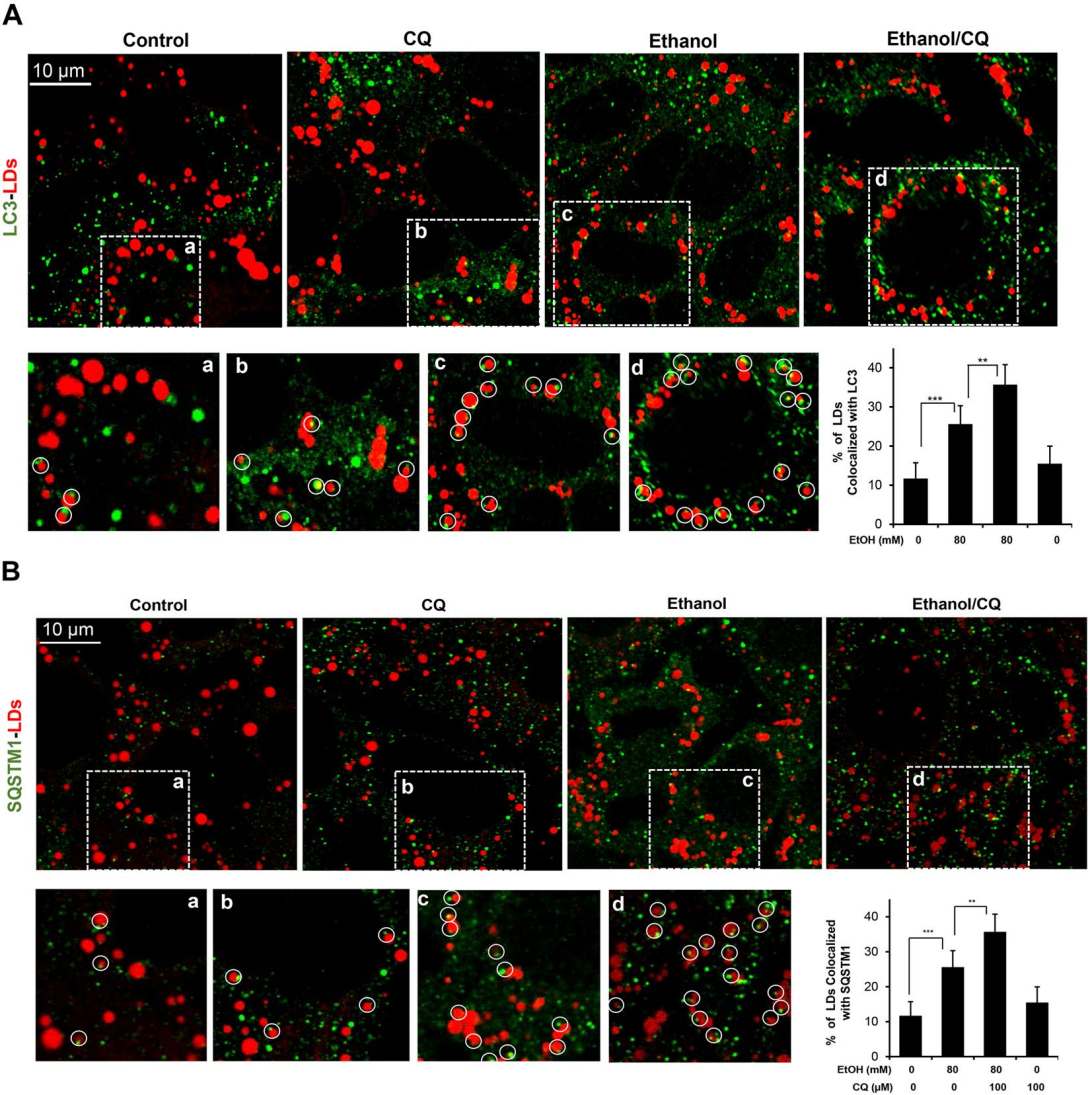
Both LC3 and SQSTM1 are found on lipid droplets. AML12 cells were treated with ethanol for 24 hours in the presence or absence of CQ in the last three hours. Cells were then stained for LC3 (**A**) or SQSTM1 (**B**), followed by staining for LDs. Representative confocal images were shown. Boxed areas (a–d) are enlarged. Lipid droplets that were colocalized with LC3 (**A**) or SQSTM1 (**B**) were circled and quantified. Data represent mean ± SEM. ^**^ *P* < 0.01, ^***^ *P* < 0.001.

The ability of autophagy to regulate lipid content in ethanol-treated AML12 cells suggested that this system was a valid model for studying autophagy-mediated lipid degradation, i.e., lipophagy with a pathophysiological significance. To understand how autophagosomes may remove lipids, we first examined whether autophagosomes can be found in the LDs. As shown in Fig. 1E, the level of LDs was regulated by autophagy. We thus examined whether autophagosomes could be found in LDs in response to ethanol treatment. Using anti-LC3 immunostaining and a Bodipy dye together with confocal microscopy, we found that the endogenous LC3 was colocalized with LDs (Fig. 3A). LC3 signals were found at the edge of LDs, and there could be more than one LC3 puncta on a single LD. The percentage of LDs with colocalized LC3 puncta was increased from about 10% to about 25% in response to ethanol treatment, which could be further elevated to above 30% in the presence of CQ, when the autophagic degradation was blocked.

To investigate how autophagosomes were recruited to the LDs, we examined whether SQSTM1, the autophagy adaptor whose level was changed during ethanol treatment and autophagy modulation (see above), could be also found on the LDs. Anti-SQSTM1 staining together with staining for LDs revealed that SQSTM1 was found on LDs in a way stimulated by ethanol and modulated by CQ, just like LC3 (Fig. 3B). Moreover, LC3, SQSTM1 and LD were colocalized together following ethanol treatment, and the signals of LC3 and SQSTM1 were merged together by confocal microscopy (Supplementary Fig. 3S), suggesting that autophagosomes could be recruited to the LDs via SQSTM1, which serves as the bridge between the autophagosome via its interaction with LC3 and also via its interaction with LDs. This model of SQSTM1-mediated selective removal of a particular target by autophagy has been shown in numerous other cases^22,23^.

**Figure 4:**
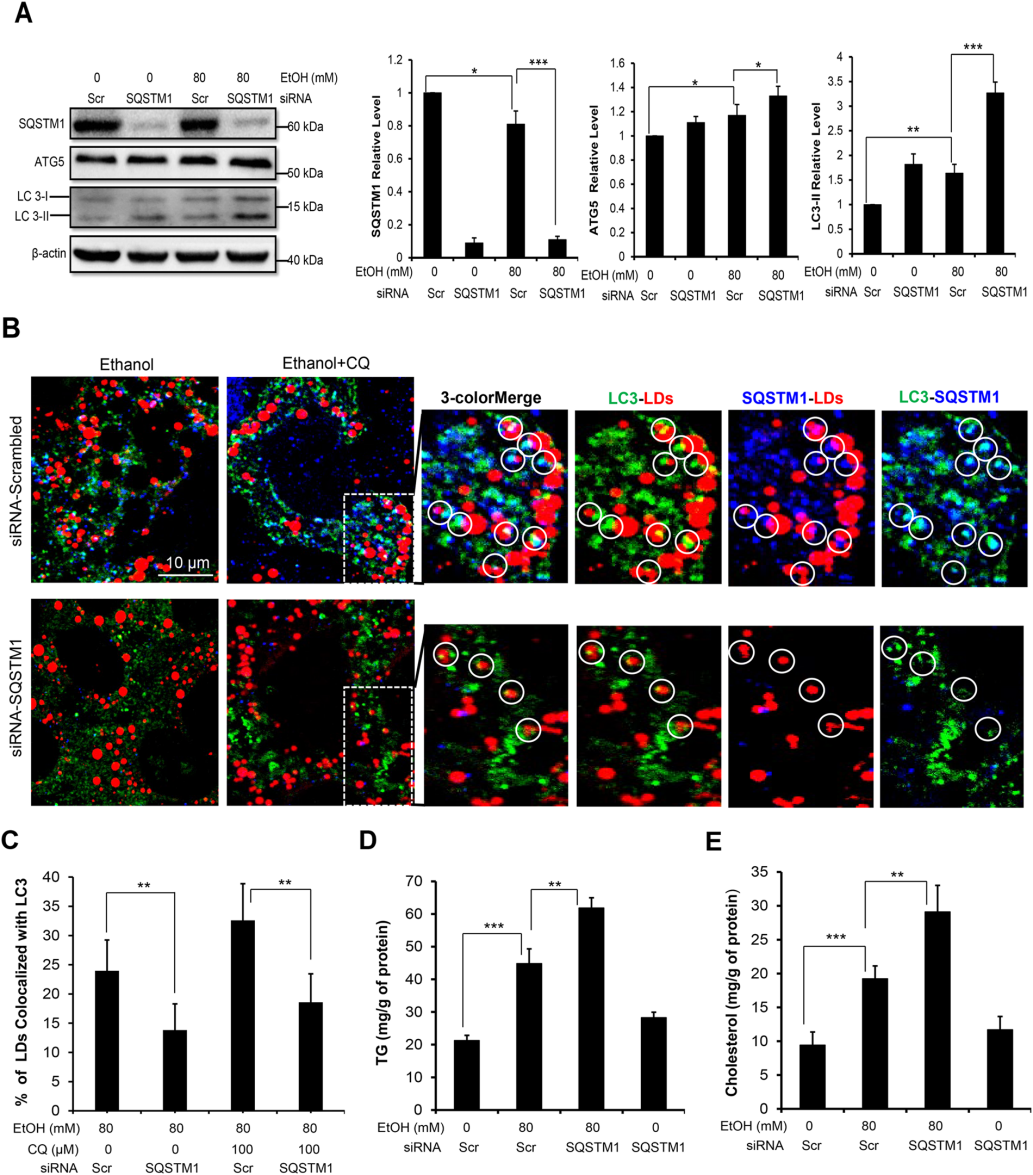
SQSTM1 is important for LC3 to be associated with LDs and ethanol-induced lipophagy. (**A**) AML12 cells were transfected with scrambled (Scr) siRNA or SQSTM1-specific siRNA, followed by treatment with ethanol plus/minus CQ for 24 hours before being analyzed by immunoblotting and densitometry. The protein levels were normalized to that of β-actin and expressed as fold of the control level. (**B** and **C**) AML12 cells were transfected with scrambled (Scr) siRNA or SQSTM1-specific siRNA, treated with ethanol for 24 hours and then stained for LC3, SQSTM1 and LDs. Representative confocal images with two or three-color merge were shown (**B**). Paired co-localizations of LC3, SQSTM1 and LDs were individually illustrated for the boxed areas of the ethanol/CQ-treated samples. The percent of LDs with co-localized LC3 signals was determined (**C**). (**D** and **E**) AML12 cells subjected to SQSTM1 knockdown as described above were collected to determine the intracellular levels of TG (**D**) and cholesterol (**E**). Data represent mean ± SEM. ^*^ *P* < 0.05, ^**^ *P* < 0.01, ^***^ *P* < 0.001.

To test this model in the present case, we knocked down SQSTM1 in AML12 cells, which resulted in an elevation of LC3 in the basal level and in the ethanol treatment condition (Fig. 4A), indicating the inhibition of autophagy flux. Knockdown of SQSTM1 did not seem to enhance cell death compared to the control transfected with scrambled siRNA. Cells were then treated with ethanol in the presence or absence of CQ, and the colocalization of LC3 and SQSTM1 to LDs was assessed by confocal microscopy. We found that the SQSTM1 was significantly reduced by immunostaining in cells transfected with the SQSTM1-specific siRNA (Fig. 4B), much like what was shown by immunoblotting assay (Fig. 4A). Therefore there were few LDs that had colocalized SQSTM1 signals. Most importantly, the percentage of LDs with colocalized LC3 puncta was significantly reduced in ethanol-treated cells (Fig. 4C). The inclusion of CQ in this assay prevented the degradation of LDs and allowed the analysis on a larger amount of LDs. This result indicated that SQSTM1 played an important role in recruiting LC3/autophagosomes to the LDs. Consistently with this interpretation, we found that the levels of TG and cholesterol were also higher in SQSTM1-knockdown AML12 cells than the control cells following ethanol treatment (Fig. 4D and E), indicating the functional importance of SQSTM1 in autophagic removal of lipids in ethanol treatment.

**Figure 5:**
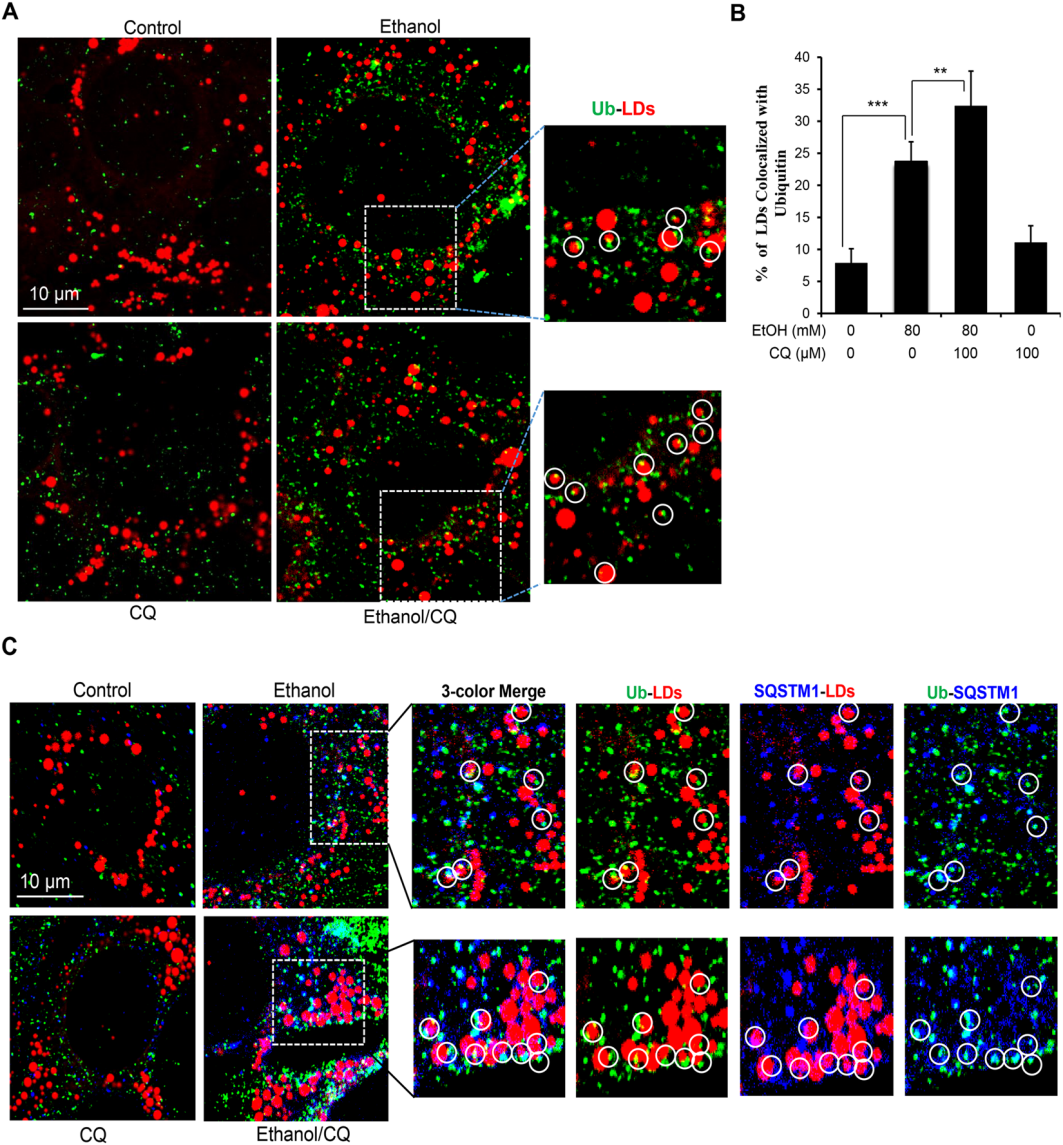
Ethanol treatment increased ubiquitin signals on lipid droplets. (**A** and **B**) AML12 cells were treated with ethanol (24 hours) and CQ (last 3 hours), and then stained with anti-ubiquitin (green) and Bodipy-581/591 (red). Representative confocal images are shown (**A**). Boxed areas are enlarged. Lipid droplets with colocalized ubiquitin (Ub) signals are circled (**A**) and quantified (**B**). (**C**) AML12 cells were treated as in **A**, and then sequentially stained for Ub, SQSTM1 and LDs. Representative confocal images with three-color merge are shown. Paired co-localizations of ubiquitin, SQSTM1 and LDs are individually illustrated for the boxed areas of the ethanol, and ethanol/CQ-treated samples. Data represent mean ± SEM. ^**^ *P* < 0.01, ^***^ *P* < 0.001.

While binding to LC3 on the autophagosome, SQSTM1 also binds to ubiquitinated cargo, thus facilitating the engulfment of the cargo by the autophagosome^22,23^. We speculated that increased ubiquitination of proteins on LDs could provide anchoring sites for SQSTM1. We examined this hypothesis by anti-ubiquitin immunostaining and found that ubiquitin signals were increased on LDs following ethanol treatment (Fig. 5A and B). Co-treatment with CQ further enhanced the percentage of LDs colocalized with ubiquitin signals. Like the LC3 and SQSTM1 signals, the ubiquitin signals were also punctated and located at the edge of the LDs. Confocal microscopy confirmed that the signals of ubiquitin and SQSTM1 overlapped on LDs (Fig. 5C), supporting the hypothesis that SQSTM1 binds to ubiquitinated targets. Taken together, these data supported the conclusion that SQSTM1 acted as an important adaptor in ethanol-induced lipophagy.

### Perilipin 1 played an important role in ethanol-induced lipophagy

Lipid droplets are composed of a neutral lipid core consisting mainly of triacylglycerols and cholesteryl esters surrounded by a phospholipid monolayer^24^. The surface of LDs is decorated by a number of proteins that are involved in the regulation of lipid metabolism. To look for the putative targets bound by SQSTM1, we first examined the proteins of the perilipin family because they are among the best characterized LD proteins with high abundance^24,25^. In the initial evaluation, we found that the distribution pattern on LDs of three perilipin family proteins, i.e., perilipin 1 (PLIN), perilipin 2 (ADRP) and perilipin 3 (TIP47), were quite different in AML12 cells (Supplementary Fig. 4S). The staining pattern of PLIN was punctated whereas those of ADRP and TIP47 were patchy and diffusive. It seemed that the distribution pattern of PLIN was most consistent with that of LC3 and SQSTM1. We thus decided to examine whether PLIN was involved in SQSTM1-mediated lipophagy in ethanol-treated AML12 cells.

**Figure 6:**
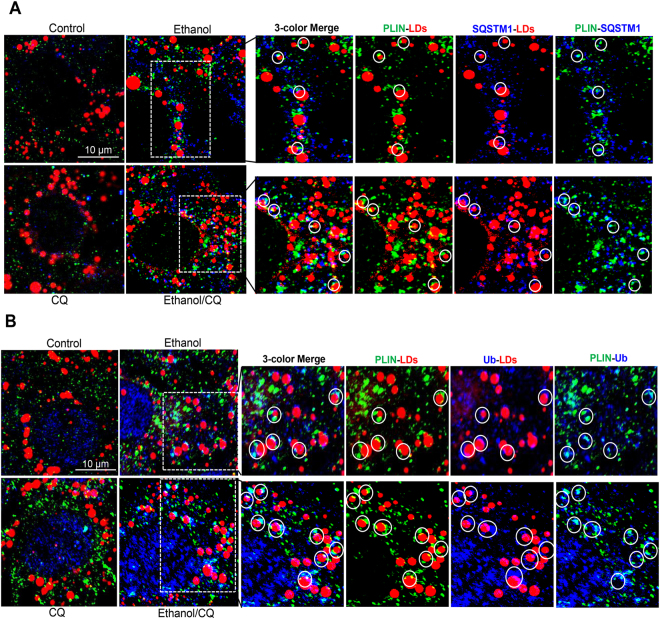
Perilipin 1 (PLIN) is colocalized with the autophagy machinery and ubiquitin signals. AML12 cells were treated with ethanol (24 hours) and CQ (last 3 hours), and then sequentially stained for PLIN, SQSTM1 and LDs (**A**), or sequentially stained for PLIN, ubiquitin (Ub) and LDs (**B**). Representative confocal images with three-color merge are shown. Paired co-localizations of PLIN, SQSTM1 with LDs (**A**) or PLIN, Ub with LDs (**B**) are illustrated for the boxed areas of the ethanol and ethanol/CQ-treated samples.

Confocal microscopy indicated that ethanol treatment increased the colocalization of PLIN and SQSTM1 on LDs (Fig. 6A). In addition, the signal of PLIN was also colocalized with that of ubiquitin on the LDs (Fig. 6B). The co-treatment of CQ blocked the degradation of LDs, resulting in more LDs in which such colocalization could be observed. These results suggested that SQSTM1 could interact with PLIN, which might be ubiquitinated, following ethanol treatment.

**Figure 7:**
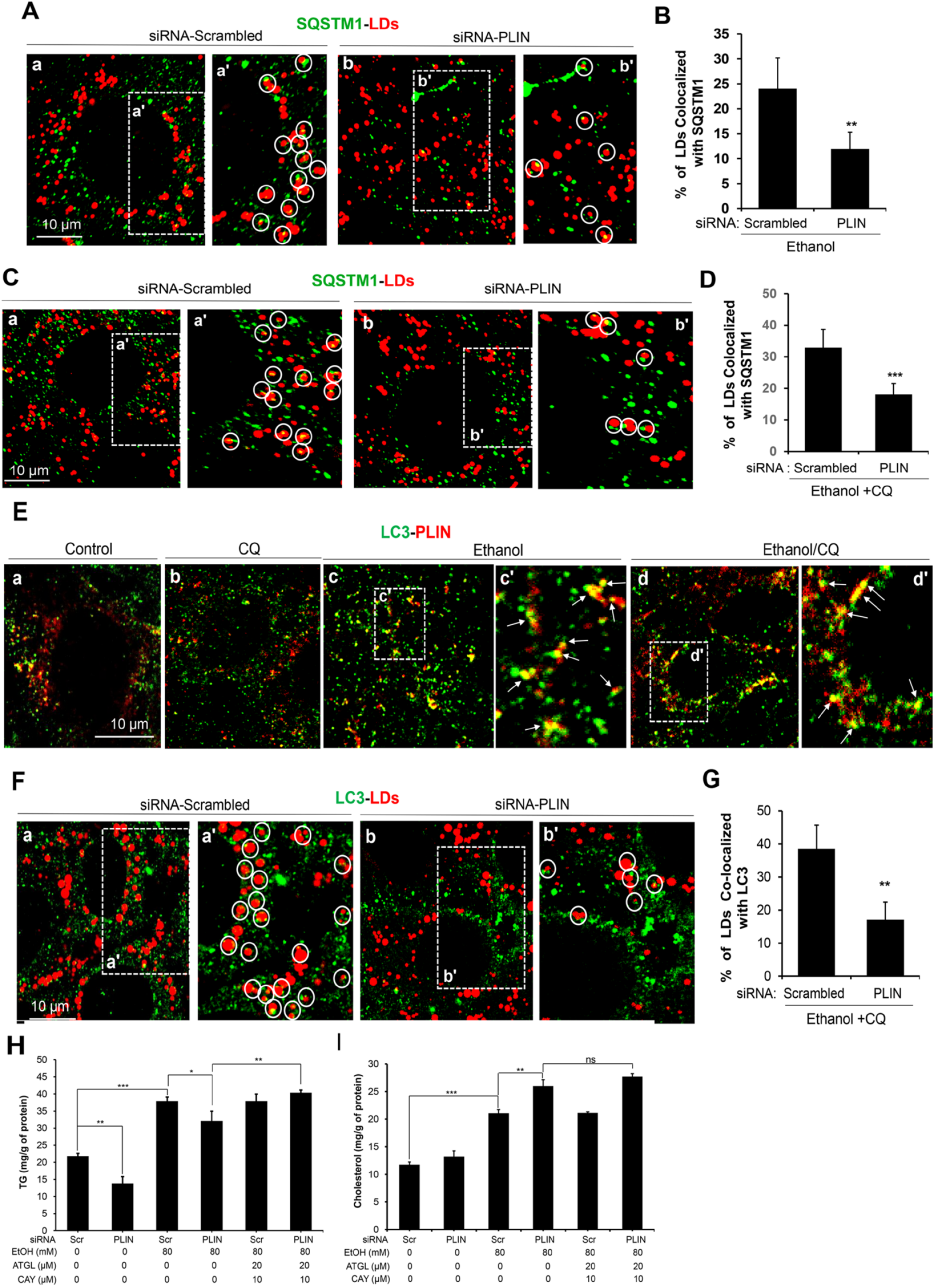
PLIN plays an important role in ethanol-induced lipophagy. (**A**–**D**) AML12 cells were transfected with scrambled (Scr) siRNA or PLIN-specific siRNA for 24 h, followed by treatment with ethanol (24 hours) alone (**A** and **B**) or in the presence of CQ (**C** and **D**), and then stained for SQSTM1 and lipid droplets. Representative confocal images (a–b) and the enlarged boxed areas (a’–b’) are shown (**A,C**). LDs with SQSTM1 signals are circled (**A**,**C)** and quantified (**B**,**D**). (**E**) AML12 cells were treated with ethanol alone or in the presence of CQ as in A, and then stained for LC3 and PLIN. Representative confocal images (a–d) with the enlarged boxed areas (c’ and d’) are shown. Arrows indicate the colocalized signals. (**F**,**G**) After transfection with siRNA as in A, AML12 cells were treated with ethanol and CQ, followed by staining for LC3 and lipid droplets. Representative confocal images (a–b) and the enlarged boxed areas (a’–b’) are shown (**F**). LDs with colocalized LC3 signals are circled (**A**) and quantified (**G**). (**H** and **I**) After transfection with siRNA as in A, AML12 cells were treated with ethanol and Atglistatin (ATGL) or CAY10499 (CAY) for 24 h. Cells were then harvested and the intracellular levels of TG (**H**) and cholesterol (**I**) were measured. Data represent mean ± SEM. ^*^ *P* < 0.05, ^**^ *P* < 0.01, ^***^ *P* < 0.001, *ns*:not significant.

To further investigate the potential role of PLIN in recruiting autophagosomes to the LDs, we selectively knocked down PLIN. The protein expression was effectively inhibited as confirmed by immunostaining (Supplementary Fig. 5SA) and immunoblotting assays (Supplementary Fig. 5SB). In addition, the SQSTM1 level was elevated significantly following PLIN knockdown, although the level of LC3 was only mildly affected (Supplementary Fig. 5SC,D). This suggested that PLIN knockdown could reduce SQSTM1 turnover. Furthermore, the colocalization of SQSTM1 to the LDs was significantly reduced by the inhibition of PLIN expression in either ethanol alone (Fig. 7A and B) or ethanol plus CQ (Fig. 7C and D) condition. This suggested that PLIN could play a significant role in recruiting SQSTM1 to the LDs, which was required for the autophagic turnover of LDs and SQSTM1. Consistently, we found that LC3 colocalized with PLIN following ethanol treatment (Fig. 7E), but the association of LC3 to the LDs was significantly inhibited by the suppression of PLIN expression (Fig. 7F and G). These results supported the notion that PLIN could serve as an anchoring site for autophagosomes via SQSTM1.

We then investigated whether knockdown of PLIN had any functional significance on lipophagy in ethanol-treated AML12 cells. However, this approach was complicated by the fact that the presence of PLIN on LDs prevents the hydrolysis of esterified lipids by lipases^25,26^. Knockdown of PLIN itself reduced the TG level, indicating the activation of the lipolysis (Fig. 7H **)**. While ethanol treatment caused TG elevation, simultaneous inhibition of PLIN expression did not result in an additional elevation of TG (Fig. 7H) as seen in the case of Atg5 knockdown (Fig. 2D) or SQSTM1 knockdown (Fig. 4D). On the other hand, PLIN knockdown elevated the level of cholesterol whether in the presence or absence of ethanol (Fig. 7I), which would be anticipated based on the result of Atg5 knockdown (Fig. 2E) and SQSTM1 knockdown (Fig. 4E). Thus PLIN likely participated in lipophagy as ATG5 and SQSTM1, but the measurement of TG level as an indication of lipophagy was compromised by the now activated lipase-mediated hydrolysis.

To alleviate the interference by the lipases, we used Atglistatin and CAY10499, inhibitors for adipose triglyceride lipase and hormone-sensitive lipase, respectively. These lipases are known to be responsible for TG hydrolysis on lipid droplets; this hydrolysis is inhibited by perilipins^25,26^. The inclusion of these two inhibitors indeed resulted in a further elevation of TG in ethanol-treated PLIN-knocked down AML12 cells (Fig. 7H). A minor but statistically insignificant effect on cholesterol level was also observed (Fig. 7I). Overall, the data supported that PLIN participated in the ethanol-triggered lipid degradation mediated by the SQSTM1-directed selective autophagy pathway.

## Discussion

We have established a cellular model to study ethanol-induced lipophagy. Using this model, we had the following findings: (1) Autophagy is activated by ethanol in AML12 cells, which requires ethanol to be oxidized; (2) Autophagy regulates the level of lipid content in ethanol-treated cells, consistent with previous *in vivo* studies that autophagy modulators affect hepatic lipid levels; (3) Autophagic degradation of lipid droplets is indicated by the colocalization of autophagosomes with the lipid droplets and the blockage of the degradation by lysosome inhibitors; (4) Recognition of lipid droplets by autophagosomes is mediated by SQSTM1, and involves ubiquitination signaling; and (5) PLIN could be a target recognized by SQSTM1 and serve as an anchor for autophagosomes on lipid droplets.

Although lipophagy has been well documented in the literature^5,6,16^, its mechanism is still far from clear. One of the major hurdles is the lack of proper cellular models to dissect and define the molecules that are involved. While it is essential to use genetically altered mice to prove a specific molecular pathway, animal models cannot be easily manipulated for events occurring at the intracellular levels. It is thus important to establish a cellular model in which ethanol can be metabolized and ethanol-induced lipid accumulation can be demonstrated.

While it may be the best to use primary hepatocytes for such purposes, primary cells have the practical concern in preparation, the difficulty in genetic manipulation and the quick loss of responsiveness to ethanol. Thus established hepatocyte lines are often used in the *in vitro* analysis. In this study, we used AML12 cells rather than other possible cell lines such as VL17 cells because AML12 cells are immortalized, but not tumorous, and naturally retain the ability to metabolize ethanol^18^. In addition, the dose of ethanol used in this study (80 mM) was not toxic to AML12 cells, although suppression of autophagy with CQ, 3-MA or Atg5 knockdown could enhance the toxicity (Wang L. unpublished observations). Furthermore, VL17 cells are derived from the human hepatocellular carcinoma cell line HepG2 with engineered ADH and Cy2E1 expression to provide it the ability to catabolize ethanol^19^. Autophagy regulation and selective autophagy function can be quite different in cancer cells comparing to that in non-cancer cells^27^. It is possible that the autophagy response of AML12 cells is close to that of normal hepatocytes. Indeed we were able to define features of ethanol-induced lipophagy in AML12 cells, which are consistent with the *in vivo* findings^5,6^. This included the demonstration of the ability of autophagy to regulate ethanol-induced lipid accumulation and changes in lipid contents. The AML12 cell line thus offers a reliable and feasible system for the study of ethanol-induced lipophagy.

Lipophagy is a type of selective autophagy. Ethanol-induced lipophagy is likely subjected to the same regulations that affect the autophagy initiation machinery in general, and that involve ethanol metabolism in particular, such as ROS^5^, and acetaldehyde^28^. Additional mechanisms, such as that mediated by TFEB, has been reported in ethanol-induced autophagy^29,30^ and can thus be also important for ethanol-induced lipophagy, particularly at the degradation step through lysosome activation. However, more specific regulation of lipophagy would involve the recognition of LDs by autophagosomes as in other types of selective autophagy. What might be different from other selective autophagy, such as mitophagy or pexophagy, is that lipophagy may not necessarily lead to the engulfment of an entire LD in a single autophagosome. Multiple autophagosomes may attack one LD, and only a piece of one LD may be taken up by one autophagosome at a time. Nevertheless, the first step of recognition by autophagosomes may still involve similar mechanisms. This recognition requires adaptor molecules, which bind to both target molecules and autophagosomes^22^. SQSTM1 is one of the better studied adaptor molecules and binds to the autophagosome via interaction with LC3 on one hand. On the other hand, SQSTM1 binds to an ubiquitinated molecule on the autophagic target, thus facilitating the engulfment of the target by the autophagosome. We have shown here that SQSTM1 is significantly involved in ethanol-induced lipophagy because knockdown of SQSTM1 significantly blocked the targeting of autophagosomes to lipid droplets and elevated the lipid content in the ethanol-treated cells. In addition, we detected increased ubiquitination on the lipid droplets and demonstrated the signals to be colocalized with SQSTM1. Notably, the classical selective autophagy is dependent on ubiquitin^23^, and we have found increased ubiquitination signals on the lipid droplets, which are colocalized with SQSTM1. Furthermore, these signals were affected by ethanol exposure and by the lysosome function, indicating that they were regulated by ethanol-induced lipophagy.

There are a very limited number of proteins present on the lipid droplets^24,25^. The most abundant proteins are the perilipins. Perilipin 1 (PLIN) has an interesting distribution on lipid droplets in AML12 cells, displaying a punctate pattern, instead of a patched or a ring-shaped pattern (Supplementary Fig. 4S). Notably, PLIN puncta could colocalize with the ubiquitin and SQSTM1 signals. These findings support the hypothesis that PLIN could serve as a target of ethanol-induced lipophagy in AML12 cells although we do not have biochemical evidence to indicate that PLIN is indeed ubiquitinated and the ubiquitinated PLIN binds to SQSTM1. We were not able to enrich PLIN in isolated lipid droplets to perform the required analysis. Additional biochemical tools are needed to further this line of study. However, we were able to provide additional genetic evidence that knockdown of PLIN reduced lipid content in ethanol-treated cells. It is noted that a major normal function of PLIN is to prevent LDs from hydrolysis^25,26^. Thus knocking down PLIN would cause a reduction in TG, which may be indistinguishable from the result of autophagic degradation. By inhibiting triglyceride hydrolysis, the impact of PLIN on lipophagy was revealed.

It is important to note that this study only implicated the role of PLIN in ethanol-induced lipophagy in AML12 cells. It is possible that other proteins, including other perilipins, on lipid droplets can be the target(s) of autophagosomes. Future studies would use multiple genetic and biochemical approaches to define the interactions and participations of additional molecules in the lipophagic process. It is also important to note that lipophagy would require molecular mechanisms not just in target recognition, but also in engulfment, transportation, and fusion with the lysosomes. Recently Rab7^31,32^ and dynamin 2^33^ have been found to play roles in starvation induced lipophagy in hepatocytes^31,33^ and in beta-adrenergic receptor-stimulated lipophagy in adipocytes^32^.

In conclusion, we have defined the importance of SQSTM1 in lipophagy triggered by ethanol in a non-tumorous cellular model that retains the ability to metabolize ethanol. This system would allow future studies to more precisely define the molecular pathway for lipophagy. The molecular mechanisms can then be examined in the *in vivo* system to verify their pathophysiologic roles, as lipophagy could be a major mechanism used by autophagy to protect against ethanol-mediated toxicity in the liver.

## Methods

### Chemicals and antibodies

All chemicals were of highest grade purity available. The following chemicals were used: ethanol (100%, Decon Labs, product number: 2716), chloroquine (CQ) (Signa-Aldrich, C6628, 100 µM), 3-Methyladenine (3-MA) (Signa-Aldrich, M9281, 2 mM), rapamycin (Selleck, S1039, 10 µM), wortmannin (Selleck, S2758, 100 nM), 4-Methylpyrazole hydrochloride (4-MP) (Signa-Aldrich, M1387, 4 mM), N-Acetyl-L-cysteine (NAC) (Sigma-Aldrich, A7250, 20 mM), Carbamazepine (CBZ) (Sigma-Aldrich, 94496, 50 µM), Atglistatin (inhibitor of adipose triglyceride lipase) (Cayman Chemicals, HY-15859, 20 µM), CAY10499 (inhibitor of hormone-sensitive lipase) (Cayman Chemicals, 10007875, 10 µM) and Bodipy-581/591 dye (Invitrogen, D-2228, 1 µM). The following primary antibodies were used: anti-LC3B (Sigma, L7543) for immunostaining (1:150 dilution), rabbit polyclonal anti-LC3^5^ for immunoblotting (1:500 dilution), anti-SQSTM1/p62 (Abnova, H00008878-M01) for immunostaining (1:100 dilution) and immunoblotting (1:2000 dilution), anti-perilipin 1 (Cell Signaling Technology, 9349) for immunoblotting (1:1000 dilution), anti-ubiquitin (P4D1) (Santa Cruz Biotechnology, sc-8017) for immunostaining (1:100 dilution), anti-Atg5 (Nanotools, 0262-100) for immunoblotting (1:200 dilution), and anti-β-actin (Sigma-Aldrich, A5441) for immunoblotting (1:5000 dilution). The secondary antibodies for immunoblotting are conjugated to horseradish peroxidase (Jackson Immuno Research, 705-505-303 and 111-006-062). For immunostaining, Alexa-488-conjugated goat anti-rabbit secondary antibody (A-11034), Alexa-488-conjugated goat anti-mouse secondary antibody (A-21121), and Alexa-405 goat anti-mouse secondary antibody (A-31553) were purchased from Thermo Fisher Scientific-Invitrogen.

### Cell culture and visualization of lipid droplets by Bodipy staining

AML12 cell line was obtained from ATCC. AML12 cells were seeded into 6-well plates (at a density of 2.0 × 10^5^ cells per well) or sterile cover glasses placed in the 24-well plates (at a density of 1.0 × 10^5^ cells per well) and cultured in DMEM/F_12_ medium supplemented with 0.005 mg/mL insulin, 0.005 mg/mL transferrin, 5 ng/mL selenium, 40 ng/mL dexamethasone, 100 U/ml of penicillin, 100 µg/ml of streptomycin, 0.25 g/L of glutamine and 10% fetal bovine serum (heat-inactivated at 56 °C) at 37 °C in the presence of 95% air and 5% CO_2_. AML12 cells cultured in sterile cover glasses placed in the 24-well plates were treated with 80 mM ethanol when it reached around 70% confluence. Ethanol treatment was repeated every 12 hours, together with any co-treatments. When AML12 cells were treated with ethanol for 21 h, 100 µM CQ was added to the medium till to the end of experiment (the action for CQ is 3 h). After the treatment, cells were fixed with 4% paraformaldehyde (PFA) for 8 min, and then stained with 1 µM Bodipy-581/591 working solution for 10 min. After being washed with PBS, coverslips were mounted and imaged on a laser scanning confocal microscope (Olympus FV1000-MPE). Bodipy-581/591 fluorescence was excited with an argon laser at 559 nm, and 50 cells were randomly selected to count the number of lipid droplets in every batch of experiment, and each one was performed in triplicate.

### Determination of TG and cholesterol levels in AML12 cells

AML12 cells were seeded in 6-well plates, and then exposed to different treatments. After the treatment, AML12 cells were collected by the trypsin digestive method, and cells in three wells were mixed together to become one sample (about 4 × 10^6^ cells in each sample). After washing the samples with PBS for 2 times, 1 mL PBS was added to each sample to make the cell suspension. Then 200 µL cell suspension was transferred to another 1.5 mL Eppendorf tube to measure the protein concentrations by the BCA method; another portion (800 µL) was used for lipid extraction. The cell pellets were re-suspended in 1 mL of chloroform-methanol mix (2:1) and incubated for 1 h at room temperature with shaking to extract the lipid. After addition of 200 µL H_2_O, samples were vortexed and centrifuged for 5 minutes at 3000* g*. The lower lipid phase was collected and dried at room temperature in a fume hood overnight. The lipid pellet was re-suspended in 60 µL of tert-butanol and 40 µL of a Triton X-114-methanol (2:1) mix. TG and cholesterol levels were quantified using the corresponding colorimetric assay kit from Pointe Scientific Inc (Canton, MI). Experimental values were standardized to the respective protein level.

### Immunoblotting analysis

After respective treatment, cells were harvested and lysed in the radio immunoprecipitation assay (RIPA) buffer supplemented with protease inhibitor cocktail. Cell lysates were centrifuged, and protein concentration was determined by BCA protein assay kit (Thermo Fisher Scientific-Pierce). Equal amounts (20 µg) of total protein were subjected to SDS-PAGE and electrotransferred to PVDF membranes. Membranes were incubated overnight at 4 °C with the primary antibodies and 1 hr with the corresponding secondary antibodies, and antibody reactions were visualized with an enhanced chemiluminescence kit (Thermo Fisher Scientific-Pierce). The signals were detected and quantified using Kodak 4000 image station and companion software. The density of each target band was normalized to that of the loading control (β-actin). Data obtained were expressed as the ratio of the intensity of the protein in the treated cells to that of the corresponding protein in control cells. Each test was performed in four different experiments with different batches of cells.

### GFP-LC3 quantification

AML12 cells were seeded in sterile glass coverslips placed in 24-well plates at a density of 1.0 × 10^5^ cells per well. When cell density reached about 50% confluence, GFP-LC3-adenovirus (diluted in PBS) was added into the medium (about 50–80 virus particles per cell) to infect AML12 cells for 12 h. 80 mM ethanol was then added into the medium for a 24-h treatment, with refreshment every 12 hours. CQ (100 µM) was added for the last 3 hours of ethanol treatment. After the treatment, cells were fixed with 4% PFA and mounted with ProLong® Gold Antifade Mountant (Life sciences, P10144). Then the coverslips were viewed under a Nikon Eclipse TE200 epi-fluorescence microscope and the companion software. At least 50 cells were randomly selected for quantification of the GFP-LC3 puncta in triplicated samples.

### Indirect immunofluorescence assay

AML12 cells were seeded on sterile glass coverslips placed in 24-well plates at a density of 1.0 × 10^5^ cells per well. After desired treatment, cells on coverslips were washed with PBS and fixed in 4% PFA for 8 min at room temperature. Fixed cells were washed with PBS and incubated with the following solution (1% Trion × 100, 15% goat serum, 15% 1 M Glycine diluted in water, 69% PBS) for 1 h at room temperature. The slides were then incubated with the primary antibodies at 4 °C overnight. After washing, the slides were incubated with the respective secondary antibodies diluted in PBS buffer for 1 h at room temperature. In the case of dual immunofluorescence staining, each set of primary and secondary antibodies was applied sequentially. When co-staining for the lipid droplets, the lipophilic dye, Bodipy-581/591 (1 µM in PBS) was applied at the end of the procedure for 10 min at room temperature. The slides were then mounted with ProLong® Gold Antifade Mountant, examined under the Olympus FV1000-MPE laser scanning confocal microscope. At least 50 cells from replicated cultures were randomly selected, and percentages of LDs with colocalized signals were quantified.

### siRNA-medicated knockdown

AML12 cells were seeded in 6-well plates at a density of 2.0 × 10^5^ cells per well. For each well, cells were transfected with 60 pmol scrambled siRNA (Invitrogen 1007792) or siRNA against Atg5 (sc-41446), p62 (sc-29828), or perilipin (sc-61323) for 6 h using 6 µL Lipofectamine RNAiMAX (Invitrogen). Subsequently the culture was refreshed with regular complete DMEM/F12 medium for another 6 h. Cells were subjected to the second transfection as described above for 12 h. Twenty-four hours after transfection, cells were treated with 80 mM ethanol or 100 µM CQ. The inhibitors of adipose triglyceride lipase (Atglistatin, 20 µM) or hormone-sensitive lipase (CAY10499, 10 µM) were included during the perilipin 1 siRNA transfection, and ethanol treatment. Treated cells were then collected to measure the levels of TG and cholesterol.

### Statistical analysis

Experiments were performed at least three times. Data are presented as the mean ± SEM of the repeated experiments. Statistical significance was determined by Student t’s analysis or one-way analysis of variance (ANOVA) (Scheffe’s post-hoc test), and *p* < 0.05 was regarded as significant.

## Electronic supplementary material

Supplemental Figures
