# Supplementary material for: Ethanol-triggered Lipophagy Requires SQSTM1 in AML12 Hepatic Cells
Source: Sci Rep. 2017 Sep 26;7:12307. doi: 10.1038/s41598-017-12485-2 (PMC5614958; doi:10.1038/s41598-017-12485-2)
Supplement: Supplementary file 1 — Supplemental Figures [file 41598_2017_12485_MOESM1_ESM.pdf]

Ethanol-triggered Lipophagy Requires SQSTM1 in AML12 Hepatic Cells

Lin Wang[1, 2, 3, 4], Jun Zhou[1, 5], Shengmin Yan[1], Guangsheng Lei[1], Chao-Hung Lee[1] and Xiao-Ming Yin[1, *]

[1] Department of Pathology and Laboratory Medicine, Indiana University School of Medicine, Indianapolis, IN, 46202, USA;
[2] College of Animal Science and Veterinary Medicine, Shandong Agricultural University, 61 Daizong Street, Tai'an City, Shandong Province, 271018, China;
[3] Shandong Provincial Key Laboratory of Animal Biotechnology and Disease Control and Prevention, Shandong Agricultural University, 61 Daizong Street, Tai'an City, Shandong Province, 271018, China;
[4] Shandong Provincial Engineering Technology Research Center of Animal Disease Control and Prevention, Shandong Agricultural University, 61 Daizong Street, Tai'an City, Shandong Province, 271018, China;
[5] Center of Minimally Invasive Surgery, Xiangya 2nd Hospital, Central South University, Changsha, Hunan, 410011, China.

Figure S1

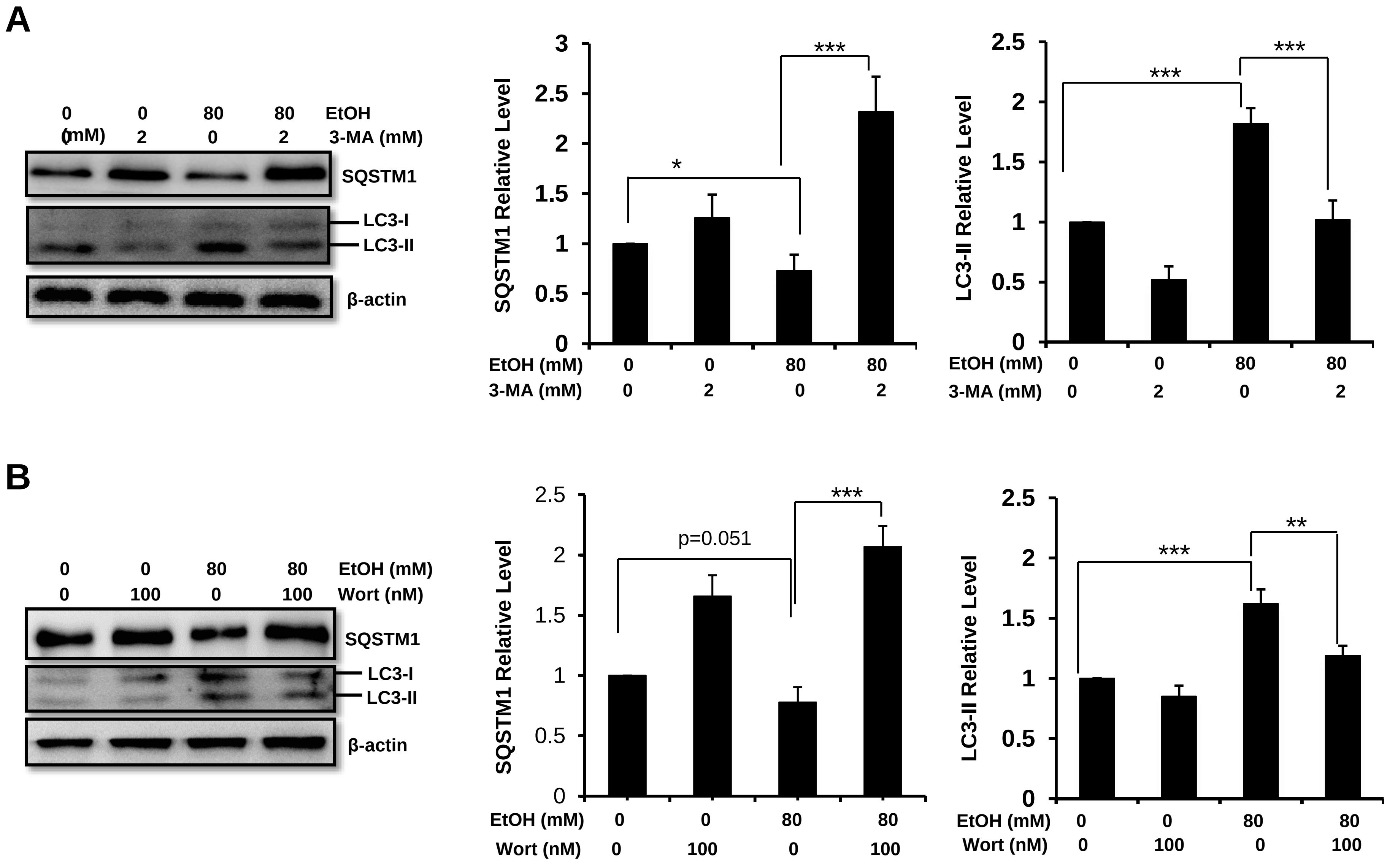
A
0
0 (mM)
0
2
80
0
80
2
EtOH
3-MA (mM)
SQSTM1
LC3-I
LC3-II
β-actin
SQSTM1 Relative Level
0
0.5
1
1.5
2
2.5
3
*
***
EtOH (mM) 0 0 80 80
3-MA (mM) 0 2 0 2
LC3-II Relative Level
0
0.5
1
1.5
2
2.5
***
***
EtOH (mM) 0 0 80 80
3-MA (mM) 0 2 0 2
B
0 0 80 80 EtOH (mM)
0 100 0 100 Wort (nM)
SQSTM1
LC3-I
LC3-II
β-actin
SQSTM1 Relative Level
0
0.5
1
1.5
2
2.5
p=0.051
***
EtOH (mM) 0 0 80 80
Wort (nM) 0 100 0 100
LC3-II Relative Level
0
0.5
1
1.5
2
2.5
***
**
EtOH (mM) 0 0 80 80
Wort (nM) 0 100 0 100

Figure S1

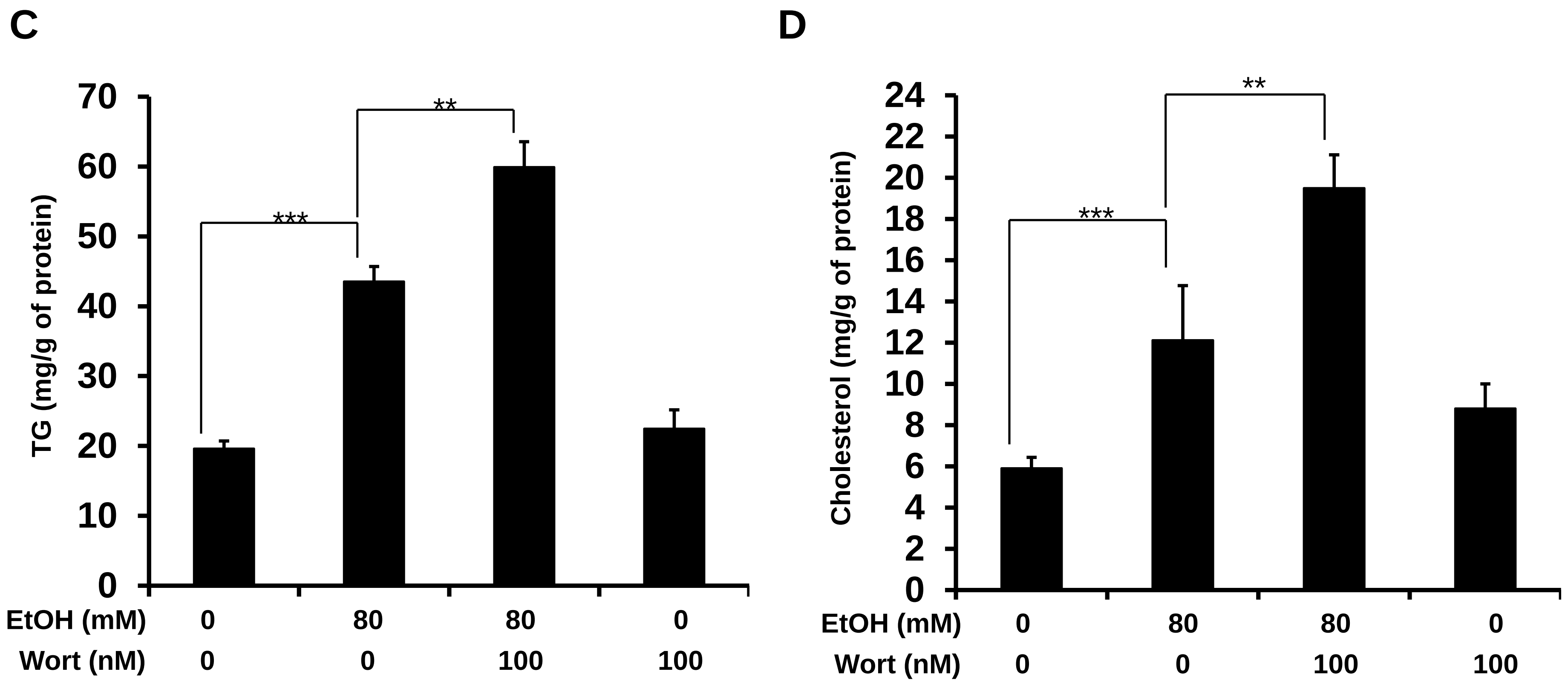

Figure 1S. Chemical inhibitors of autophagy exacerbated ethanol-induced lipid accumulation.

(**A-B**). AML12 cells were incubated with ethanol (80 mM) in the presence or absence of 3-MA (2mM, A) or wortmannin (100 nM, B) for 24 hours. Lysates were prepared and analyzed by immunoblotting and densitometry for SQSTM1/p62 and LC3. The levels were normalized to that of beta-actin and expressed as the fold of the control. (**C-D**). Cells were incubated with ethanol (80 mM) and wortmanin (100 nM) for 24 hour. Intracellular levels of TG (C) and cholesterol (D) were then determined. * $p < 0.05$, ** $p < 0.01$, *** $p < 0.001$.

Figure S2

A

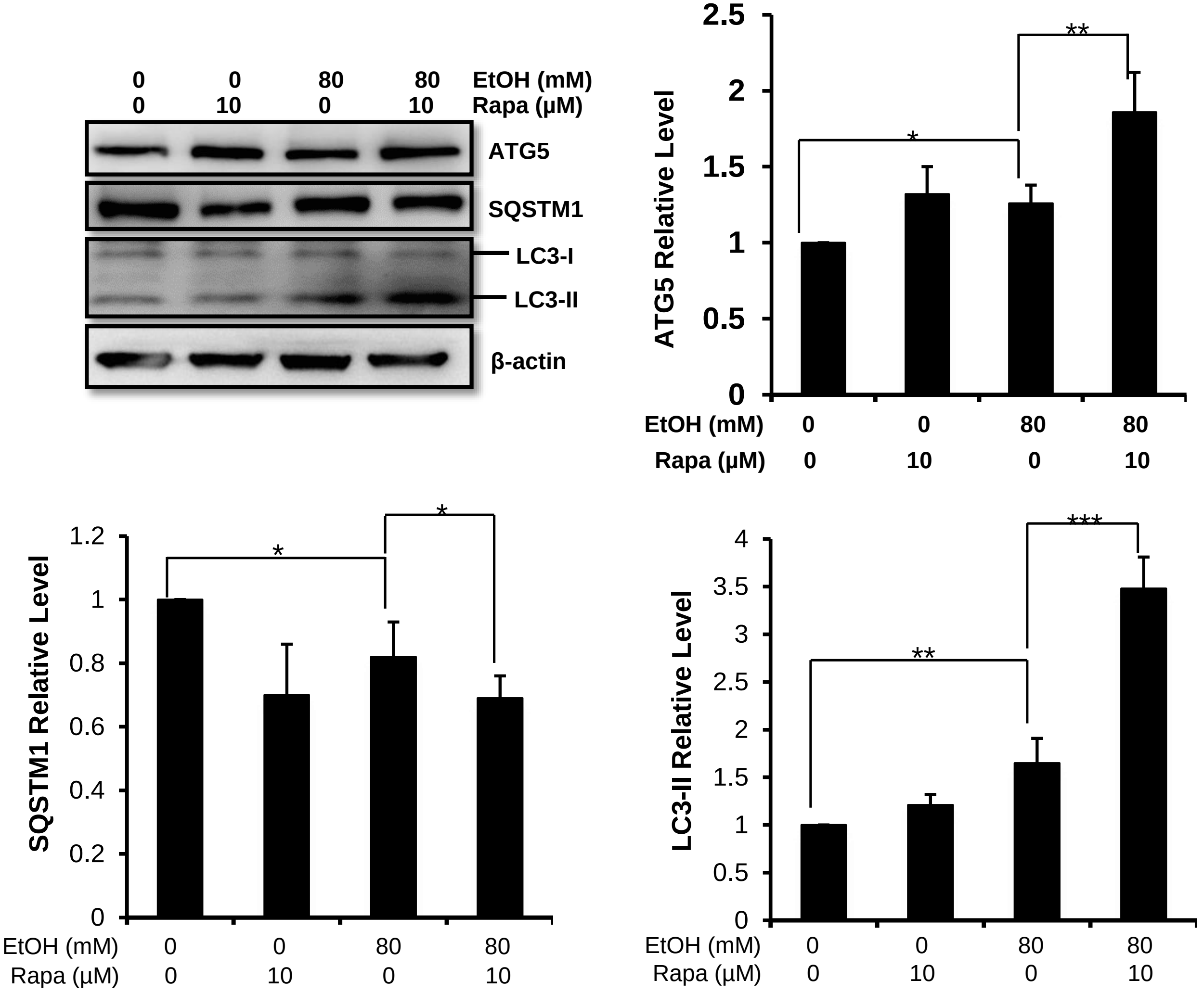
0 0 80 80 EtOH (mM)
0 10 0 10 Rapa (μM)
ATG5
SQSTM1
LC3-I
LC3-II
β-actin
ATG5 Relative Level
2.5
2
1.5
1
0.5
0
**
*
EtOH (mM) 0 0 80 80
Rapa (μM) 0 10 0 10
SQSTM1 Relative Level
1.2
1
0.8
0.6
0.4
0.2
0
*
*
EtOH (mM) 0 0 80 80
Rapa (μM) 0 10 0 10
LC3-II Relative Level
4
3.5
3
2.5
2
1.5
1
0.5
0
***
**
EtOH (mM) 0 0 80 80
Rapa (μM) 0 10 0 10

Figure S2

B

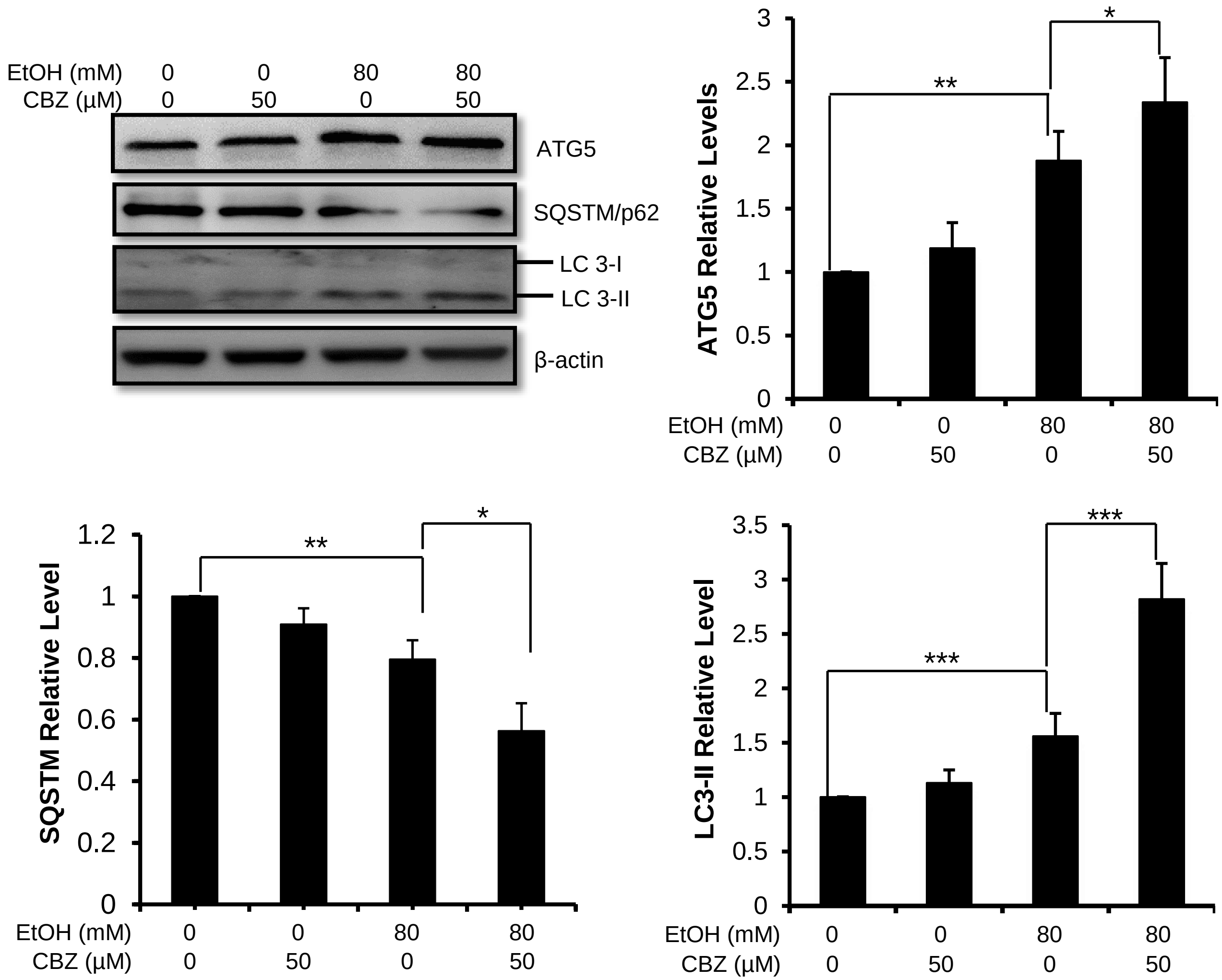

Figure 2S. Chemical activators of autophagy enhanced ethanol-induced autophagy.

AML12 cells were incubated with ethanol (80 mM) in the presence of absence of rapamycin (10 μM, **A**) or carbamazepine (CBZ, 50 μM, **B**) for 24 hours. Lysates were analyzed by immunoblotting and densitometry for ATG5, SQSTM1/p62 and LC3. The levels were normalized to that of beta-actin and expressed as the fold of the control. * $p < 0.05$, ** $p < 0.01$, *** $p < 0.001$.

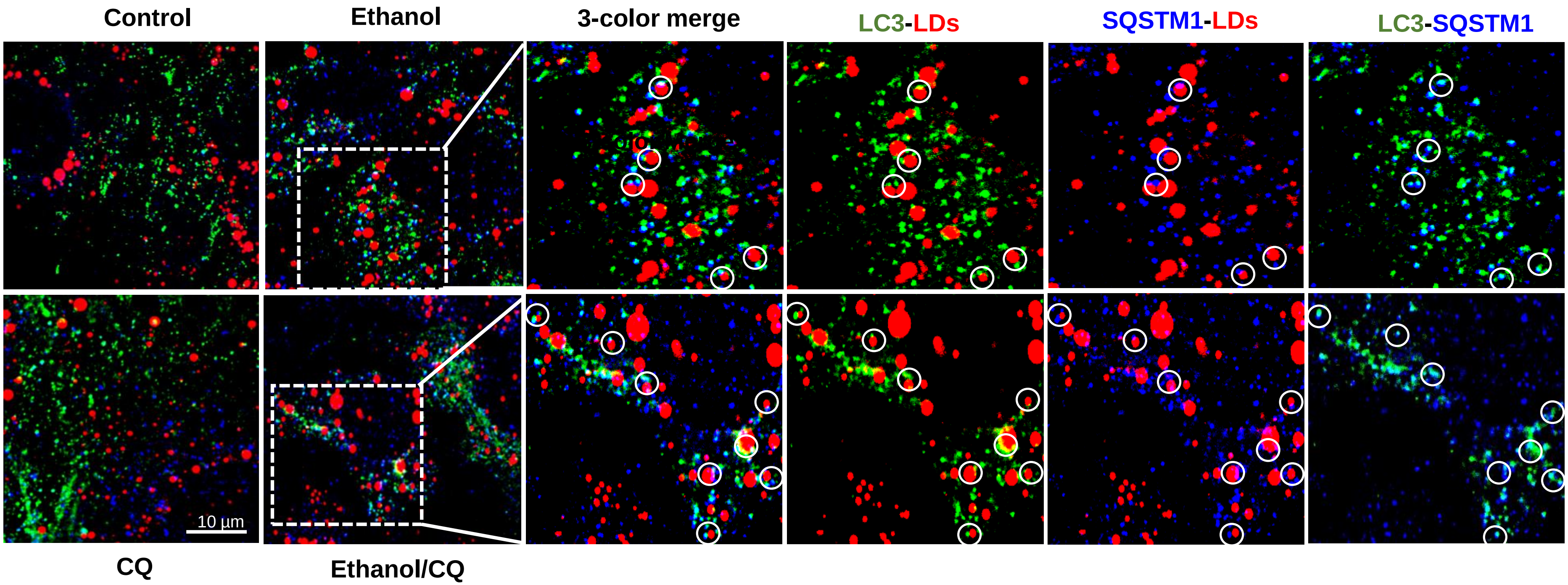

Figure 3S. Both LC3 and SQSTM1 can be found on lipid droplets following ethanol treatment.

AML12 cells were treated with 80 mM ethanol in the presence or absence of 100 µM CQ for 24 hours, then successively stained for LC3, SQSTM1 and lipid droplets. Representative confocal images with three-color merge were shown. Paired co-localizations of LC3, SQSTM1 and LDs were separately illustrated for the boxed areas of the ethanol-treated samples. Circles indicate colocalized signals.

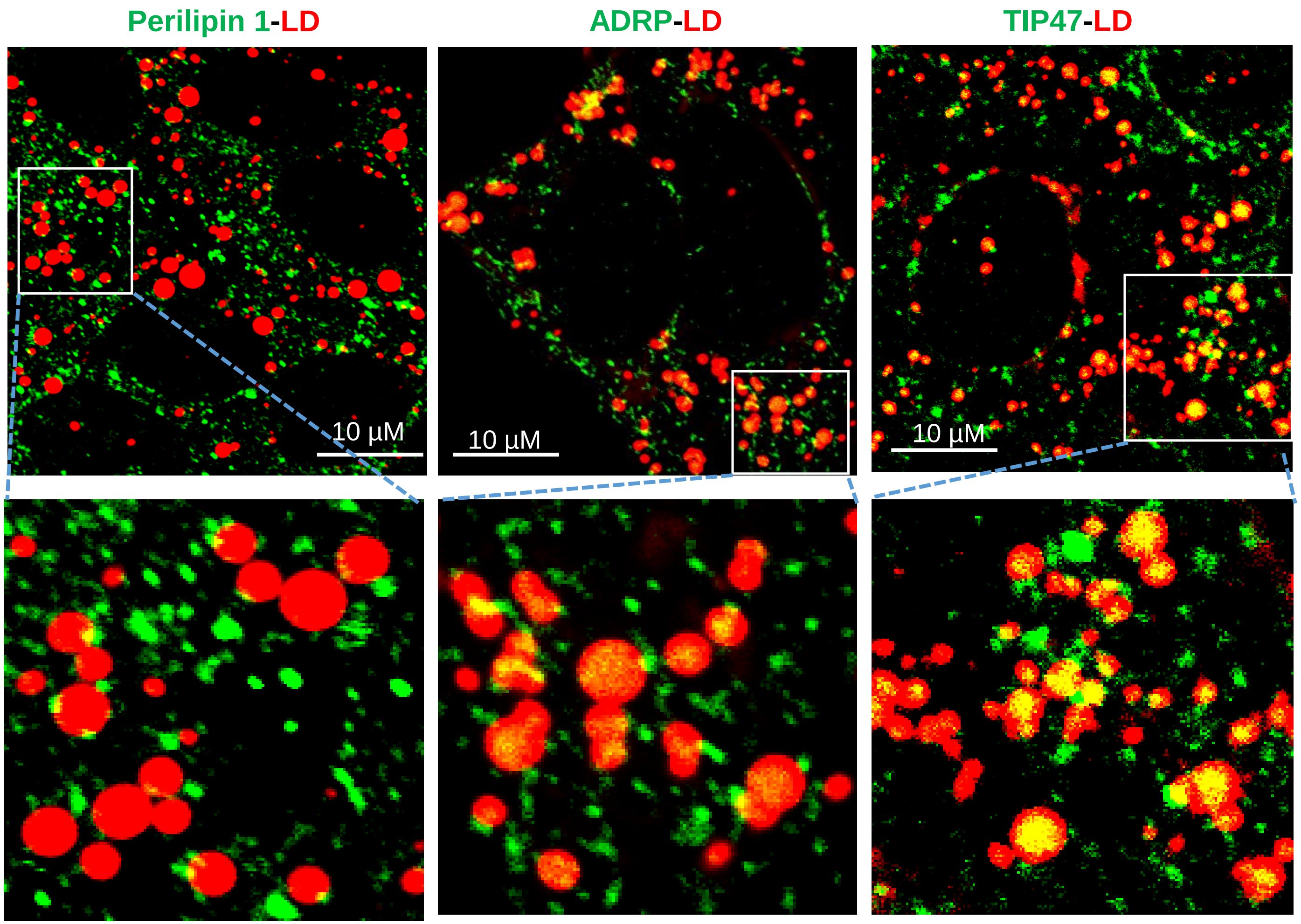

Figure 4S. **Perilipin 1 has a punctated distribution pattern on lipid droplets.**

AML12 cells were fixed, stained for perilipin-1, ADRP or TIP47 (green) respectively; followed by staining with Bodipy-581/591 for lipid droplets (red). Cells were examined by confocal microscopy. Boxed areas are enlarged in the lower panels. The staining patterns of perilipin-1, ADRP and TIP47 on the lipid droplets seem different in AML12 cells with perilipin 1 being mainly punctated, and ADRP and TIP47 being mainly patchy and diffusive.

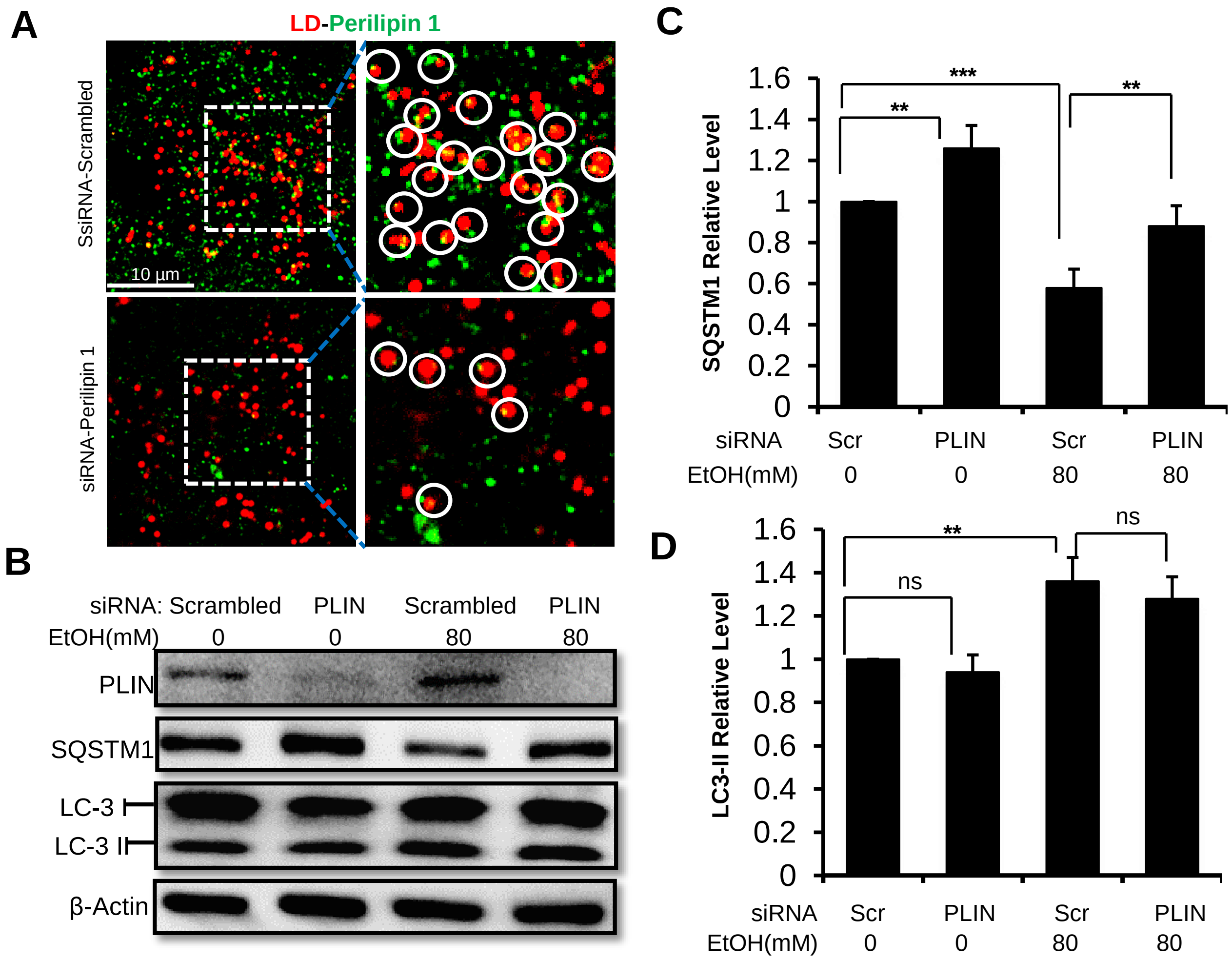

Figure 5S. Perilipin 1 can be effectively knocked down.

(**A-B**) AML12 cells were transfected with scramble (Scr) siRNA or perilipin 1 (PLIN)-specific siRNA for 24 h and examined for perilipin 1 expression by immunostaining for perilipin 1 and Bodipy-581/591 for lipid droplets (A) or immunoblotting (B). (**C-D**) The levels of SQSTM1 and LC3-II in panel B were normalized to that of β-actin and expressed as fold of the control. ** *P* < 0.01, *** *P* < 0.001. *ns* Not significant.

Figure 1

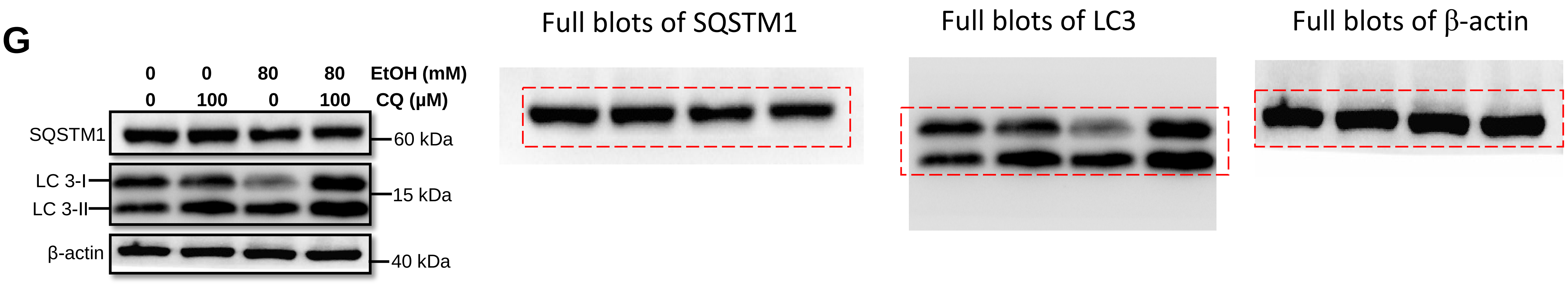
G
0 0 80 80 EtOH (mM)
0 100 0 100 CQ (μM)
SQSTM1
60 kDa
LC 3-I
LC 3-II
15 kDa
β-actin
40 kDa
Full blots of SQSTM1
Full blots of LC3
Full blots of β-actin

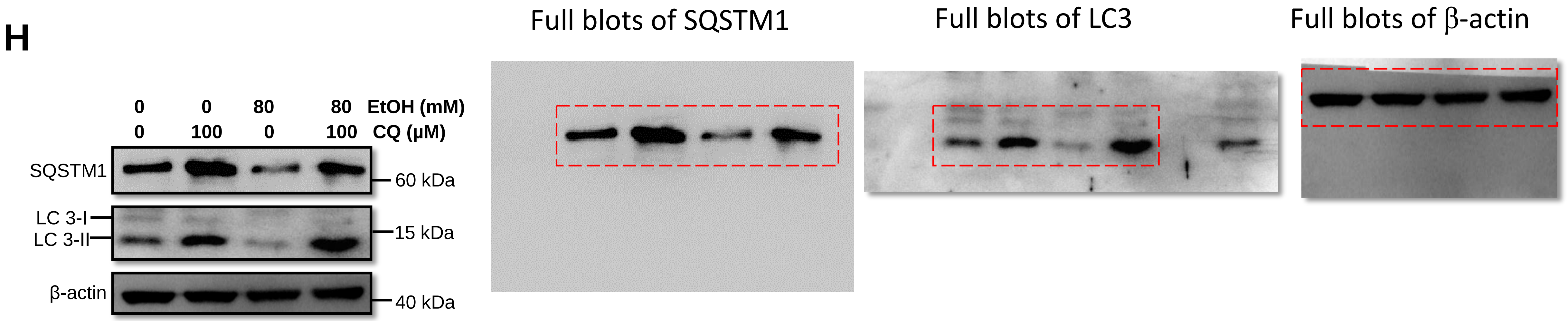
H
0 0 80 80 EtOH (mM)
0 100 0 100 CQ (μM)
SQSTM1
60 kDa
LC 3-I
LC 3-II
15 kDa
β-actin
40 kDa
Full blots of SQSTM1
Full blots of LC3
Full blots of β-actin

Figure 2

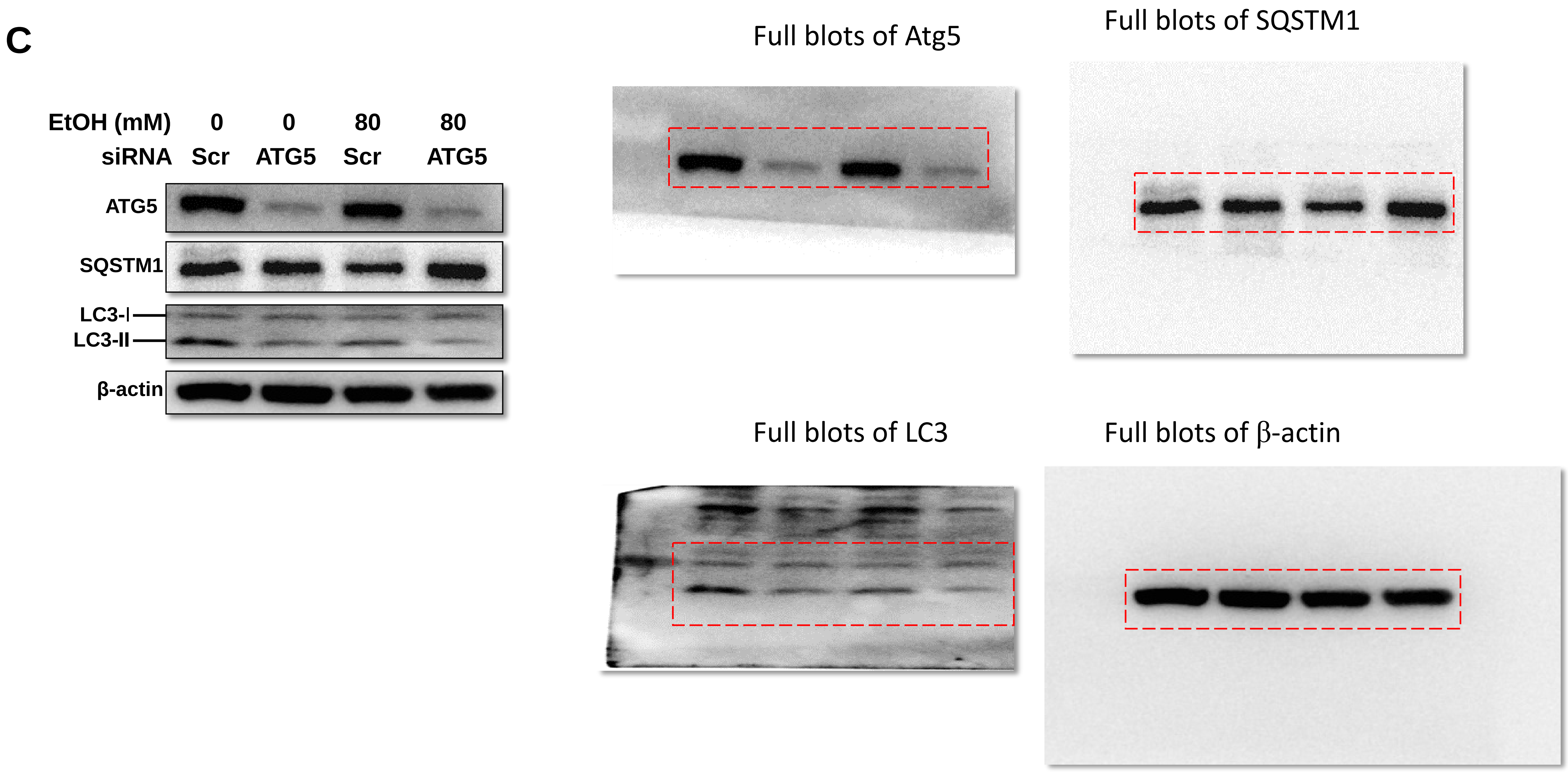
C
EtOH (mM) 0 0 80 80
siRNA Scr ATG5 Scr ATG5
ATG5
SQSTM1
LC3-I
LC3-II
β-actin
Full blots of Atg5
Full blots of SQSTM1
Full blots of LC3
Full blots of β-actin

Figure 4

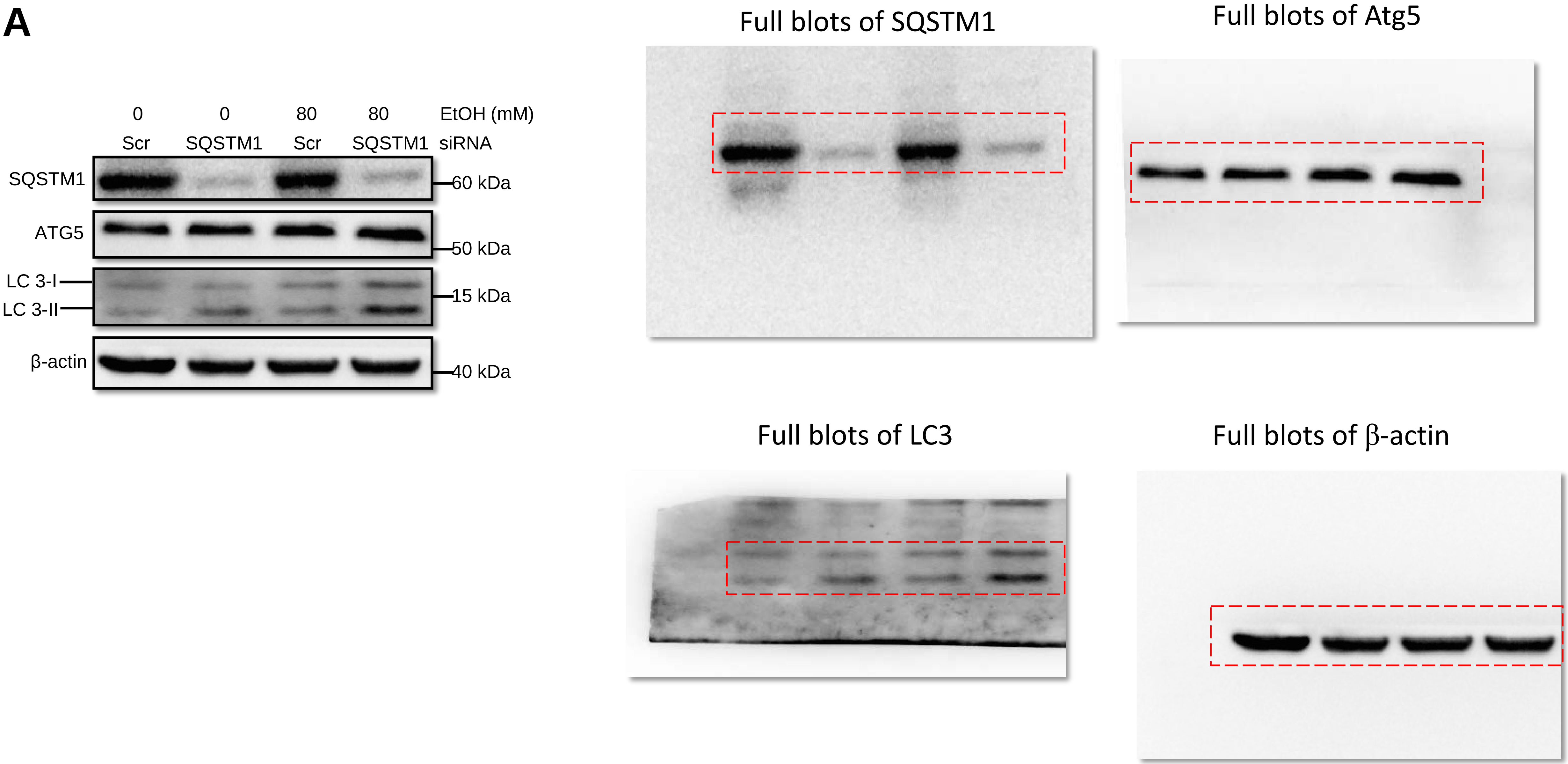
A
0
0
80
80
EtOH (mM)
Scr
SQSTM1
Scr
SQSTM1
siRNA
SQSTM1
ATG5
LC 3-I
LC 3-II
β-actin
60 kDa
50 kDa
15 kDa
40 kDa
Full blots of SQSTM1
Full blots of Atg5
Full blots of LC3
Full blots of β-actin

Figure S1

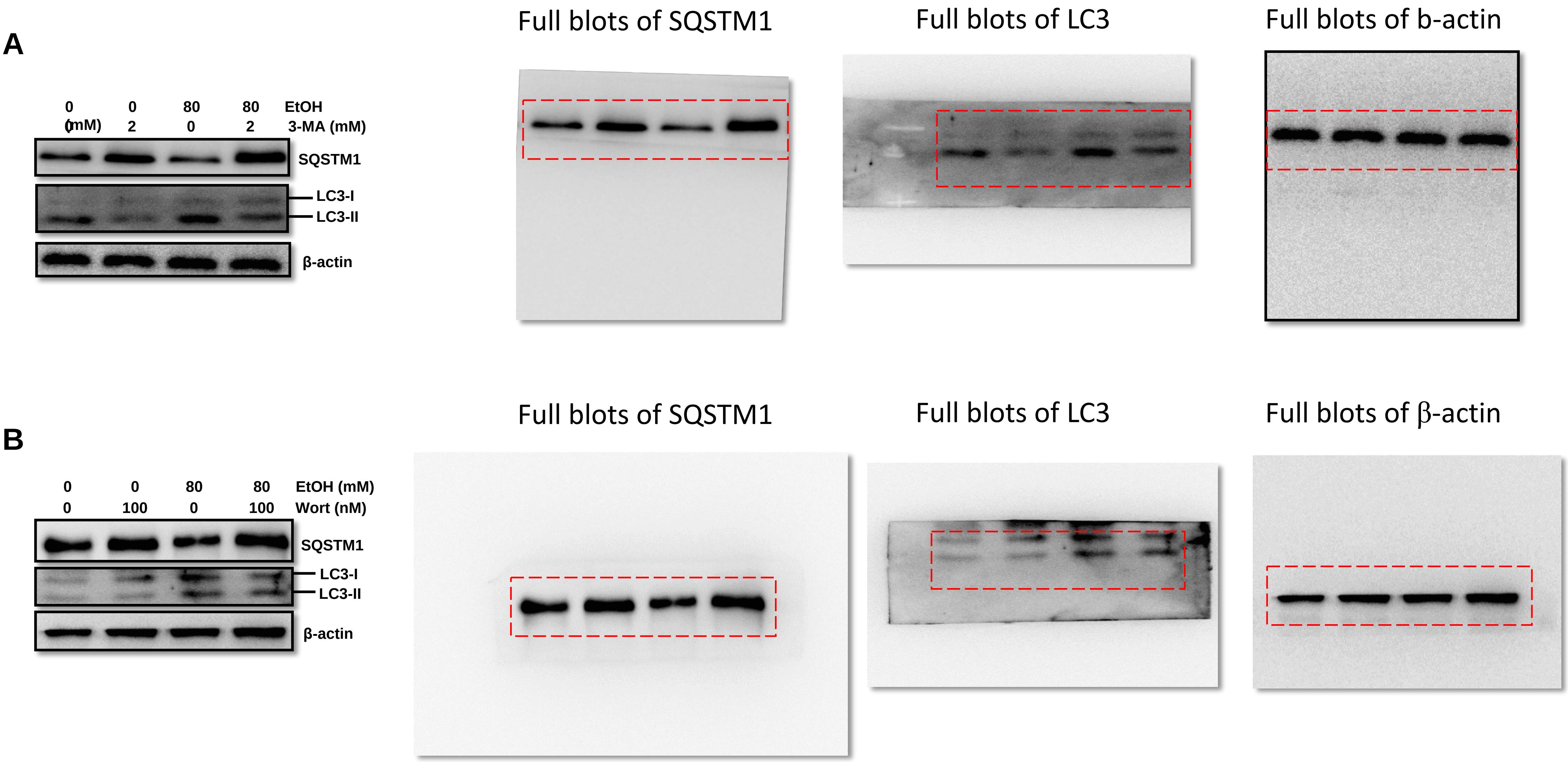
A
Full blots of SQSTM1
Full blots of LC3
Full blots of b-actin
0
0
80
80
EtOH
(mM)
2
0
2
3-MA (mM)
SQSTM1
LC3-I
LC3-II
β-actin
B
Full blots of SQSTM1
Full blots of LC3
Full blots of β-actin
0
0
80
80
EtOH (mM)
0
100
0
100
Wort (nM)
SQSTM1
LC3-I
LC3-II
β-actin

Figure S2

A

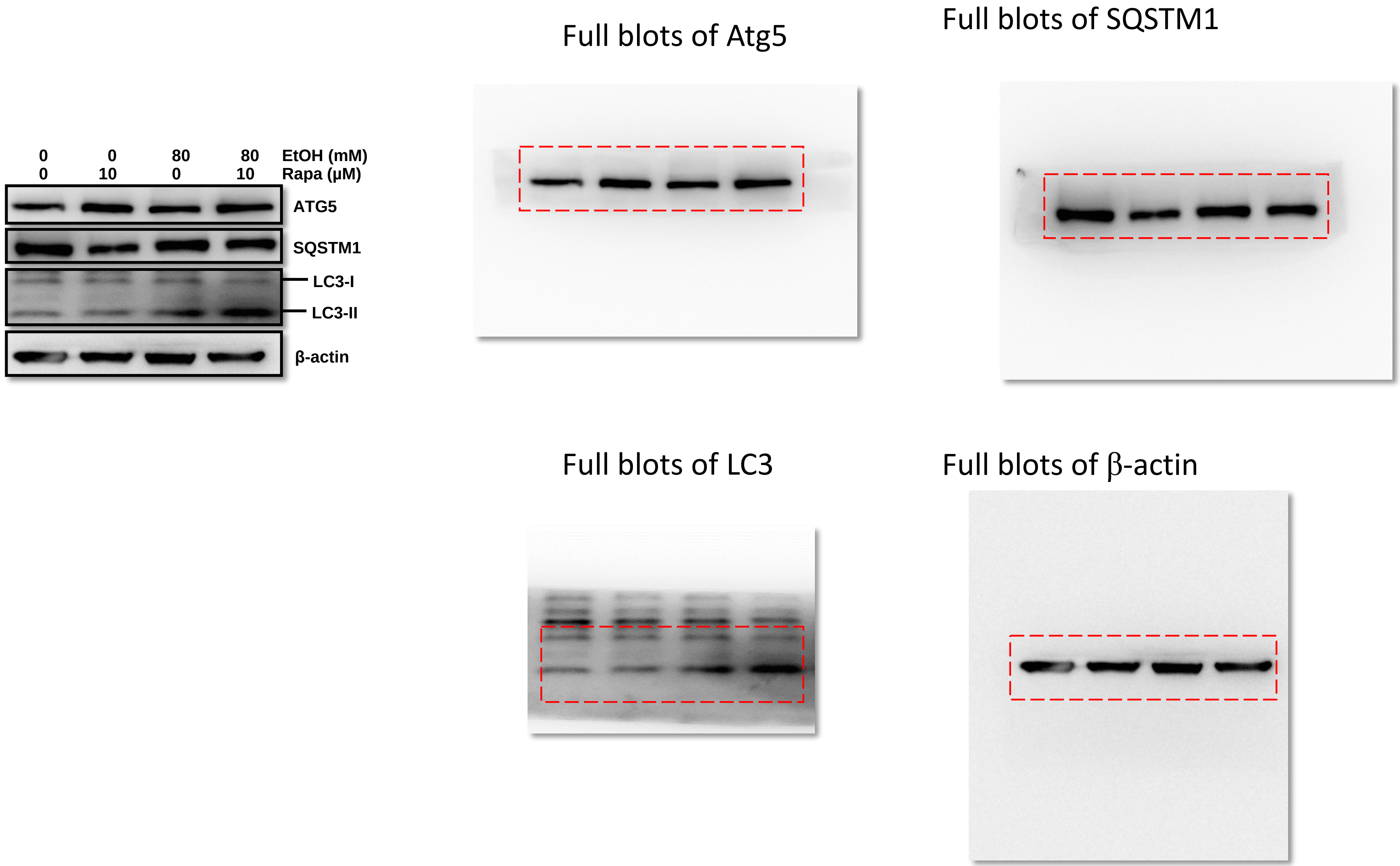

0 0 80 80 EtOH (mM)
0 10 0 10 Rapa (μM)
ATG5
SQSTM1
LC3-I
LC3-II
β-actin
Full blots of Atg5
Full blots of SQSTM1
Full blots of LC3
Full blots of β-actin

Figure S2

B

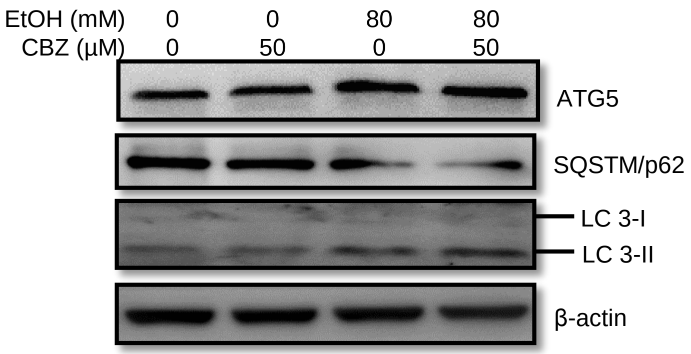

Full blots of Atg5

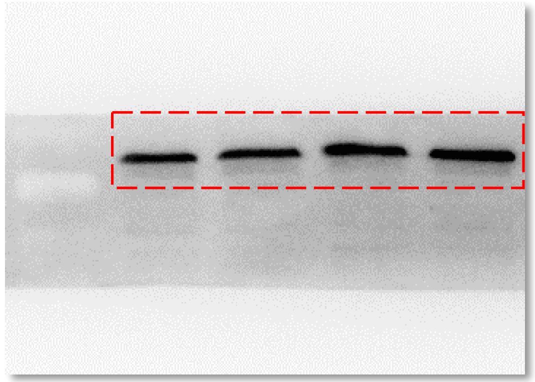

Full blots of SQSTM1

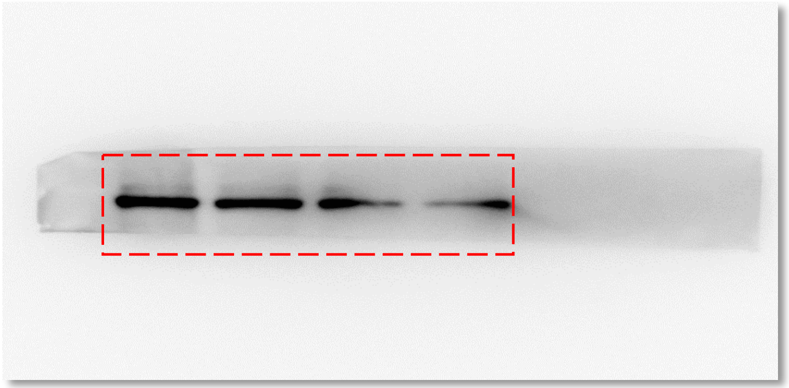

Full blots of LC3

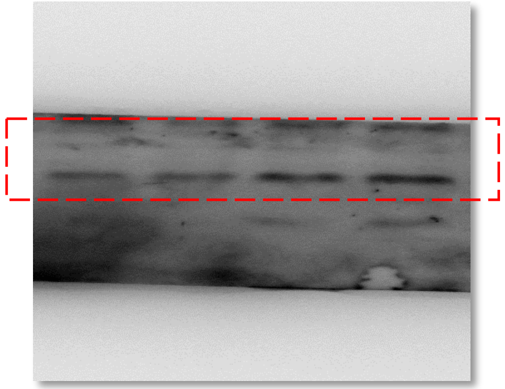

Full blots of β-actin

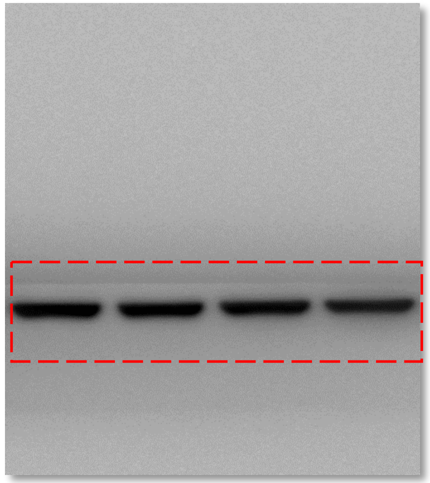

Figure S5

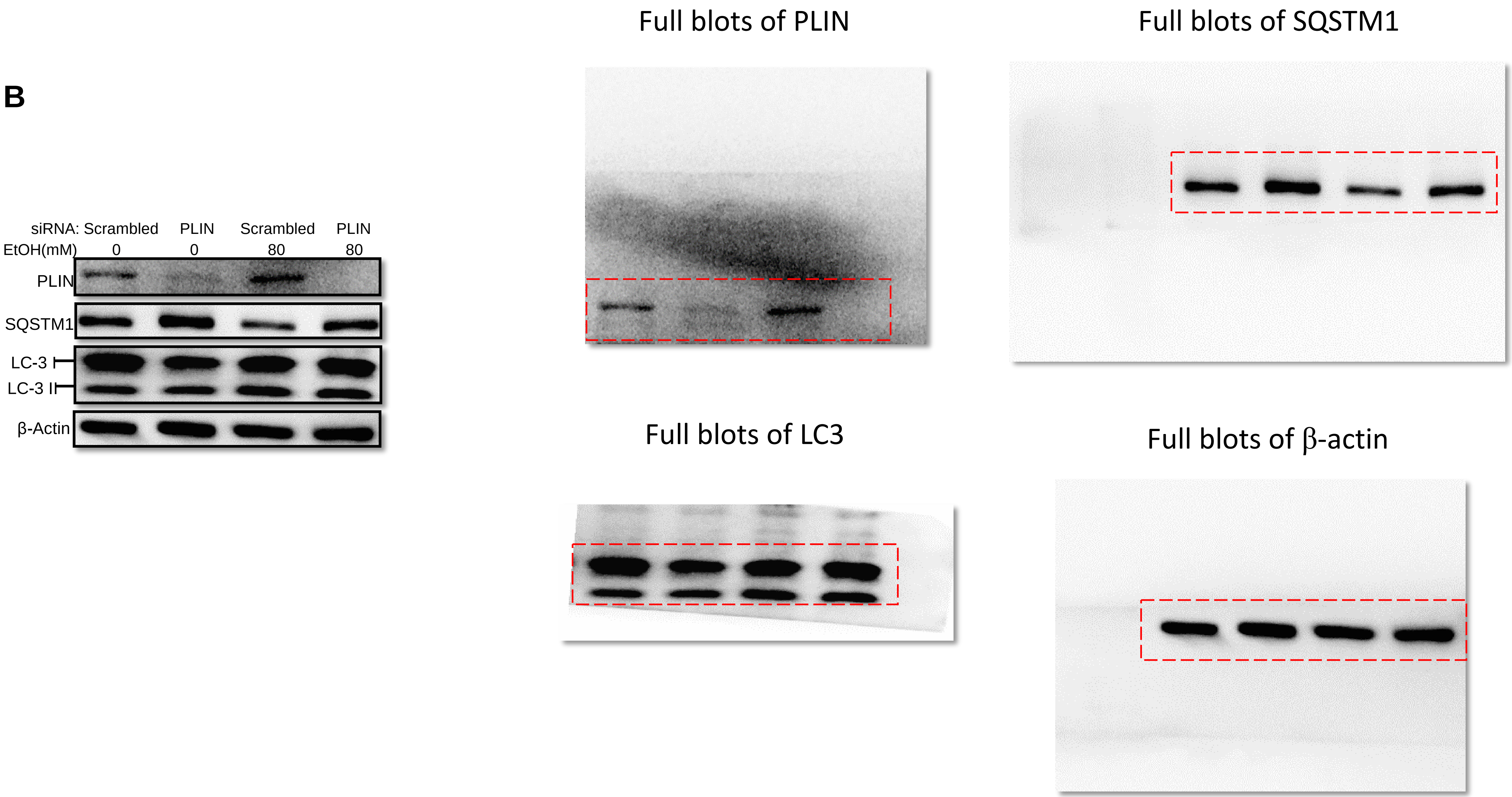

B
siRNA: Scrambled
PLIN
Scrambled
PLIN
EtOH(mM)
0
0
80
80
PLIN
SQSTM1
LC-3 I
LC-3 II
β-Actin
Full blots of PLIN
Full blots of SQSTM1
Full blots of LC3
Full blots of β-actin
